# Mitochondrial genome sequencing, mapping, and assembly benchmarking for *Culicoides* species (Diptera: Ceratopogonidae)

**DOI:** 10.1186/s12864-022-08743-x

**Published:** 2022-08-13

**Authors:** Yoamel Milián-García, Christopher A. Hempel, Lauren A. A. Janke, Robert G. Young, Tara Furukawa-Stoffer, Aruna Ambagala, Dirk Steinke, Robert H. Hanner

**Affiliations:** 1 Department of Integrative Biology, University of Guelph, 50 Stone Rd E, Guelph, ON N1G 2W1 Canada; 2 John H. Daniels Faculty of Architecture, Landscape, and Design, University of Toronto, 33 Willcocks Street, Toronto, ON M5S 3B3 Canada; 3 Canadian Food Inspection Agency, National Centre for Animal Disease, 225090 Township Road 9-1, Lethbridge LaboratoryLethbridge, AB T1J 0P3 Canada; 4 National Centre for Foreign Animal Disease, 1015, Arlington Street, Winnipeg, MB R3E 3M4 Canada

**Keywords:** Biting midges, Mitogenomics mitogenome, Vector, Diptera, High-Throughput Sequencing, mtDNA

## Abstract

**Background:**

Mitochondrial genomes are the most sequenced genomes after bacterial and fungal genomic DNA. However, little information on mitogenomes is available for multiple metazoan taxa, such as *Culicoides*, a globally distributed, megadiverse genus containing 1,347 species.

**Aim:**

Generating novel mitogenomic information from single *Culicoides sonorensis* and *C. biguttatus* specimens, comparing available mitogenome mapping and de novo assembly tools, and identifying the best performing strategy and tools for *Culicoides* species.

**Results:**

We present two novel and fully annotated mitochondrial haplotypes for two *Culicoides* species, *C. sonorensis* and *C. biguttatus*. We also annotated or re-annotated the only available reference mitogenome for *C. sonorensis* and *C. arakawae*. All species present a high similarity in mitogenome organization. The general gene arrangement for all *Culicoides* species was identical to the ancestral insect mitochondrial genome. Only short spacers were found in *C. sonorensis* (up to 30 bp), contrary to *C. biguttatus* (up to 114 bp). The mitochondrial genes ATP8, NAD2, NAD6, and LSU rRNA exhibited the highest nucleotide diversity and pairwise interspecific p genetic distance, suggesting that these genes might be suitable and complementary molecular barcodes for *Culicoides* identification in addition to the commonly utilized COI gene.

We observed performance differences between the compared mitogenome generation strategies. The mapping strategy outperformed the de novo assembly strategy, but mapping results were partially biased in the absence of species-specific reference mitogenome. Among the utilized tools, BWA performed best for *C. sonorensis* while SPAdes, MEGAHIT, and MitoZ were among the best for *C. biguttatus*. The best-performing mitogenome annotator was MITOS2. Additionally, we were able to recover exogenous mitochondrial DNA from *Bos taurus* (biting midges host) from a *C. biguttatus* blood meal sample.

**Conclusions:**

Two novel annotated mitogenome haplotypes for *C. sonorensis* and *C. biguttatus* using High-Throughput Sequencing are presented. Current results are useful as the baseline for mitogenome reconstruction of the remaining *Culicoides* species from single specimens to HTS and genome annotation. Mapping to a species-specific reference mitogenome generated better results for *Culicoides* mitochondrial genome reconstruction than de novo assembly, while de novo assembly resulted better in the absence of a closely related reference mitogenome. These results have direct implications for molecular-based identification of these vectors of human and zoonotic diseases, setting the basis for using the whole mitochondrial genome as a marker in *Culicoides* identification.

**Supplementary Information:**

The online version contains supplementary material available at 10.1186/s12864-022-08743-x.

## Background

Metazoan mitogenomes are among the most abundant genomes deposited in nucleotide databases to date [1–3], with mitochondrial markers being selected as the most common DNA barcodes for species identification [4]. The metazoan mitochondrial genome consists of extranuclear DNA of relatively small size (16 to 20 kbp) that exists in a high copy number per animal cell. Animal mitogenomes show very conserved genetic content, a limited occurrence of gene duplications, short intergenic segments, and generally lack introns [5, 6]. These features have made mitochondrial DNA (mtDNA) the most accessible genomic information for many species. Furthermore, the characteristics of mtDNA enable the analysis of complex evolutionary processes such as gene rearrangements, replication, transcription, and regulation of gene expression at a resolution that is currently not possible for nuclear genomes [6].

Most metazoan mitochondrial genomes contain 37 genes, including 22 tRNAs, 13 protein-coding genes (PCGs), and two rRNAs [6]. Mitochondrial gene products are involved in cellular energy production through oxidative phosphorylation (OXPHOS) [7]. Along with some imported products from the cytoplasm, they also allow for mitochondrial autonomy in DNA replication, transcription, and translation of organellar proteins [6]. In addition, the mitochondrial genome includes a non-coding, A-T rich control region known as the Displacement loop (D-loop), which is responsible for regulating mtDNA replication and transcription [6]. Despite gene content being preserved in most metazoans, gene arrangements and duplications can distinguish specific evolutionary lineages [6]. As gene rearrangements are generally uncommon within but vary among major lineages, their study allows for the resolution of taxonomic relationships using mitochondrial genome analysis (mitogenomics), unravelling complex evolutionary histories [6, 8]. Fragments of the mitogenomes can also be transferred into the nuclear DNA, constituting nuclear mitochondrial DNA sequences (NUMTs). Therefore, specific tests are needed to validate the mitochondrial origin of newly sequenced genomes [9]. Although mtDNA provides limited information when species diverged recently or if introgression has occurred [5], analyzing the sequence variation of specific mitochondrial markers (e.g. Cytochrome c oxidase I [COI]) enables the DNA-based identification of metazoans, as many of them exhibit higher interspecific than intraspecific genetic differentiation. This capability constitutes the major pillar of DNA-barcoding and metabarcoding.

Mitogenomes in the phylum Arthropoda are the second most studied behind Chordata [6, 10]. The mtDNAs of *Drosophila melanogaster* and *D. yakuba* were the first mitogenomes sequenced among invertebrates [11, 12], and the mitogenome organization of *D. yakuba*, among others, resembles the ancestral gene arrangement in insects [13]. According to the NCBI Reference Sequence Database (RefSeq, https://www.ncbi.nlm.nih.gov/refseq/statistics/), thousands of mitogenomes have been released almost 40 years after the first invertebrate mitochondrial genomes were sequenced. The bloom of mitogenomic information in the last decade has mainly been facilitated by the advances in High-Throughput Sequencing (HTS) technologies. However, although the sequencing capacities of such technologies enable routine sequencing of mitochondrial genomes, the full reconstruction of mitochondrial genomes is far from being routine yet, due to the low number of tools available for this task [14]. Reconstructing mitochondrial genomes from HTS data can be achieved by following two main strategies: mapping to a reference mitogenome or *de novo* assembly [3]. Mapping to a reference using tools such as BWA [15, 16], Bowtie2 [17], Bowtie [18], Minimap2 [19], BBMap [20], and Geneious [https://www.geneious.com; Biomatters Ltd.] is the faster and more straightforward approach, considering a closely related reference mitochondrial genome is available. Distantly related references can yield erroneous mitogenome reconstructions associated with a high number of errors/mismatches [3]. Therefore, *de novo* assembly through tools such as MEGAHIT [21], SPAdes [22], rnaSPAdes [23], MitoFlex [24], MitoZ [25], MITObim [26], and NOVOPlasty [3] plays an essential role in the absence of a reference or the presence of a distantly related genome. Consequently, the best strategy will rely on several factors such as target taxa, reference mitogenome availability, HTS dataset quality, and computational power [3]. HTS generates millions to billions of reads, sufficient to sequence full mitogenomes cost-effectively and with an unprecedented depth of coverage. However, current mitogenome sequencing efforts are still underrepresented in many taxa. One example of significant mitogenome underrepresentation in databases (e.g., GenBank) is the polytypic genus *Culicoides* (Diptera: Ceratopogonidae).

*Culicoides* are biting midges considered among the smallest hematophagous insects with body sizes ranging from 1–3 mm, and they are found abundantly worldwide [27]. Morphological characters can only be studied in detail with a microscope, and the lack of comprehensive taxonomic keys for all life stages hinders species identification based on morphology. There are currently 1,347 recognized species of *Culicoides* [28], but a complete and fully annotated mitochondrial genome is only available for one species (*Culicoides (Meijerehelea) arakawae* Arakawa, 1910) [29] according to the Organelle Genome Resources of NCBI (https://www.ncbi.nlm.nih.gov/genome/organelle/). After performing a comprehensive search for mitogenomic information on *Culicoides,* we were able to find one additional, non-annotated scaffold linked to the complete mitogenome of *Culicoides (Monoculicoides) sonorensis* Wirth and Jones, 1957 [30]. The current taxonomic classification of *Culicoides* species is mainly based on morphological characters, which provide limited taxonomic as well as phylogenetic resolution in several cases and can result in taxonomic uncertainty [28]. Mitogenomics is a valuable method to complement traditional taxonomy and establish an effective and consistent DNA-based molecular identification system at all life stages. It can also be a powerful method for reconstructing phylogenomic relationships among species. Out of the 1,347 known *Culicoides* species, 336 species are classified in species groups of uncertain affiliation, 136 lack taxonomic resolution, and 875 are classified to the subgenus level [28]. Across their extensive geographic range, *Culicoides* species are a vector for human, wildlife, and domestic animal pathogens that include over 50 viruses (e.g. Oropouche virus, African horse sickness virus, and bluetongue virus) that have been isolated from these biting midges [27]. Mitogenomics for *Culicoides* species offers an exceptional opportunity for inferring phylogenomic patterns among vectors, hosts, and vectored diseases; however, this approach depends on the mitochondrial genome description for the species under study. To facilitate mitogenomic studies in *Culicoides*, we generated novel mtDNA information from single specimens of two *Culicoides* species: *C. sonorensis* and *Culicoides (Silvaticulicoides) biguttatus* (Coquillett, 1901) and re-annotated the other two available in the database. As part of that process, we evaluated whether mitochondrial isolation prior to DNA extraction increased mitogenome generation efficiency and benchmarked various tools commonly used for mitogenome generation and annotation, including mapping to reference genomes of closely related species and de novo assembly.

## Results

### DNA integrity validation

**Fig. 1:**
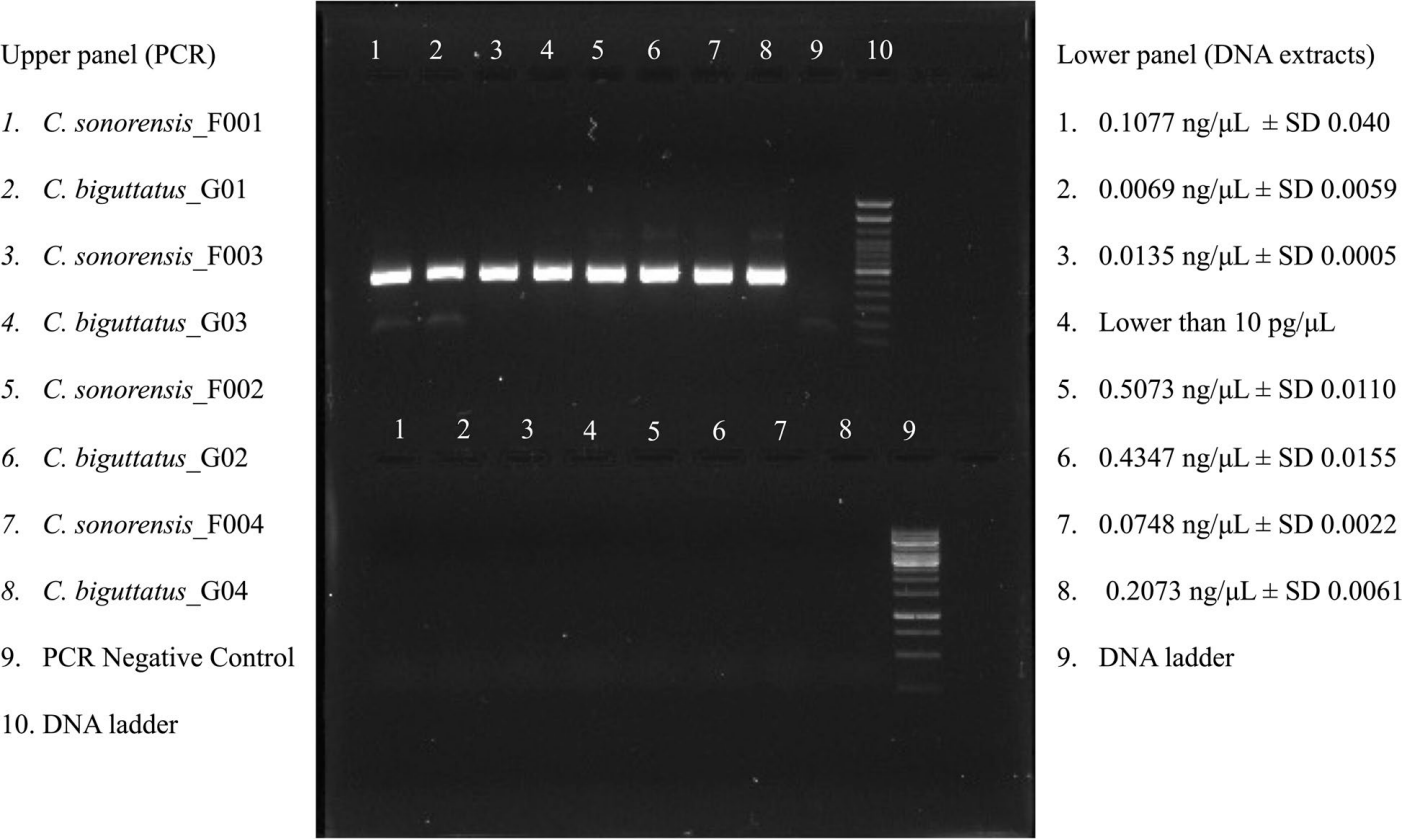
Cytochrome Oxidase I PCR amplification (Upper panel) and DNA extracts (Lower panel) of each sample run on a 1% agarose gel. Specimen names are shown on the left of the gel image, while average DNA concentrations are based on Qubit fluorometry, and their standard deviation is shown on the right of the gel image for each DNA extract. The molecular ladder represents a 100 bp DNA ladder from Thermo Scientific (catalogue number # 15628019)

DNA extractions were considered successful if DNA extract could be quantified via Qubit and the COI gene could be amplified via PCR using COI-specific primers, which was the case for all specimens and mitochondrial fractions. However, the concentrations of all extracts were low (< 1 ng/uL) (Fig. 1). Despite the success of DNA extractions, complete mitogenomes could not be generated from samples for which mitochondria were isolated prior to DNA extraction, likely due to the low amount of species-specific mitochondrial DNA extracted and consequently the low number of reads (from 4,872 to 21,356) that the respective samples received (Supplementary Table S1). Mitochondrial DNA from the *Culicoides* host *Bos taurus* was detected in specimen *C. biguttatus*_G04, ratifying the presence of exogenous DNA in the extractions (Supplementary Figure S1).

**Fig. 2:**
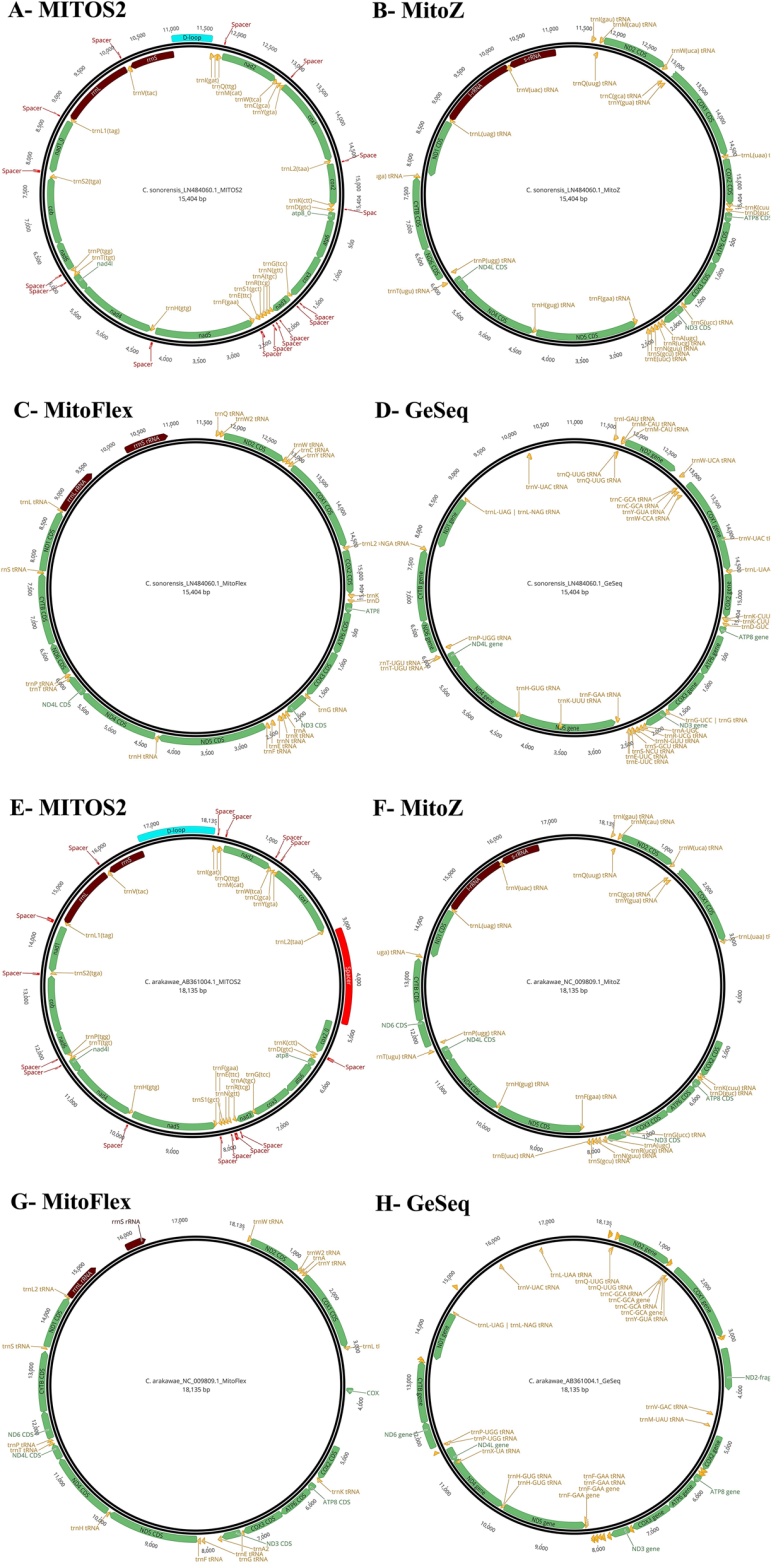
Performance of tested annotation tools (MITOS2, MitoZ, MitoFlex, and GeSeq) for the reference mitochondrial genomes of *C. sonorensis* (**A**, **B**, **C**, and **D**) and C. arakawae (**E**, **F**, **G**, and **H**). Annotation results were represented by performance, where MITOS2 had the best performance in terms of gene annotation. PCGs, rRNA, and tRNA are indicated in green, brown, and orange, respectively. The control region (D-loop) and the intergenic spacers are noted in blue and red

Among all the annotation tools, MITOS2 and MitoZ performed better than MitoFlex and GeSeq on the reference mitogenomes (*C. arakawae* and *C. sonorensis*), recovering all the 37 mitochondrial genes (13 PCGs, 22 tRNA, 2rRNA) in all the cases. GeSeq failed to identify the ribosomal coding genes (rRNAs) (Fig. 2), overestimated the number of tRNAs, or imprecisely annotated the beginning and the end of their coding region. MitoFlex performed better than GeSeq but did not identify all tRNAs and lacked the versatility of the output file, limiting the subsequent visualization of the mitogenome annotation. In addition, MitoZ, MitoFlex, and GenSeq did not include the potential origin of mtDNA replication compared to MITOS2, which was selected as the reference annotation tool moving forward.

After gene annotation, the morphological species classification of the specimens was validated via molecular identification of the species using the whole annotated COI gene via MITOS2 from the best mitogenome of each species. The top BLAST hit of each species confirmed the species-specific classification based on morphological characters (Table 1).

**Table 1:** The molecular identity of specimens used in the present study from which novel mitogenome information was generated. Molecular IDs were inferred from the reconstructed COI gene after mapping to the reference mitogenome, using the top BLAST hit from GenBank and the identification engine from BOLD. PI % – the percentage of identity; SI % – the percentage of sequence similarity. BIN ID – Barcode Index Number Identifier

GenBank							
Sample ID	COI length (bp)	Accession	Order	Family	Genus	Species	PI (%)
*C. sonorensis*_F002	1,536	LN484060.1	Diptera	Ceratopogonidae	*Culicoides*	*C. sonorensis*	99.87
*C. sonorensis*_F004	1,536	LN484060.1	Diptera	Ceratopogonidae	*Culicoides*	*C. sonorensis*	99.93
*C. biguttatus*_G02	1,539	KR656554.1	Diptera	Ceratopogonidae	*Culicoides*	*C. biguttatus*	100
*C. biguttatus*_G04	1,539	HM412494.1	Diptera	Ceratopogonidae	*Culicoides*	*C. biguttatus*	99.85
BOLD							
		BIN ID	Order	Family	Genus	Species	SI (%)
*C. sonorensis*_F002	1,536	BOLD:AAY9576	Diptera	Ceratopogonidae	*Culicoides*	*C. sonorensis*	100
*C. sonorensis*_F004	1,536	BOLD:AAY9576	Diptera	Ceratopogonidae	*Culicoides*	*C. sonorensis*	100
*C. biguttatus*_G02	1,539	BOLD:AAG6468	Diptera	Ceratopogonidae	*Culicoides*	*C. biguttatus*	100
*C. biguttatus*_G04	1,539	BOLD:AAG6468	Diptera	Ceratopogonidae	*Culicoides*	*C. biguttatus*	99.84

### Evaluation of mitogenomes based on mapping to reference genomes

**Fig. 3:**
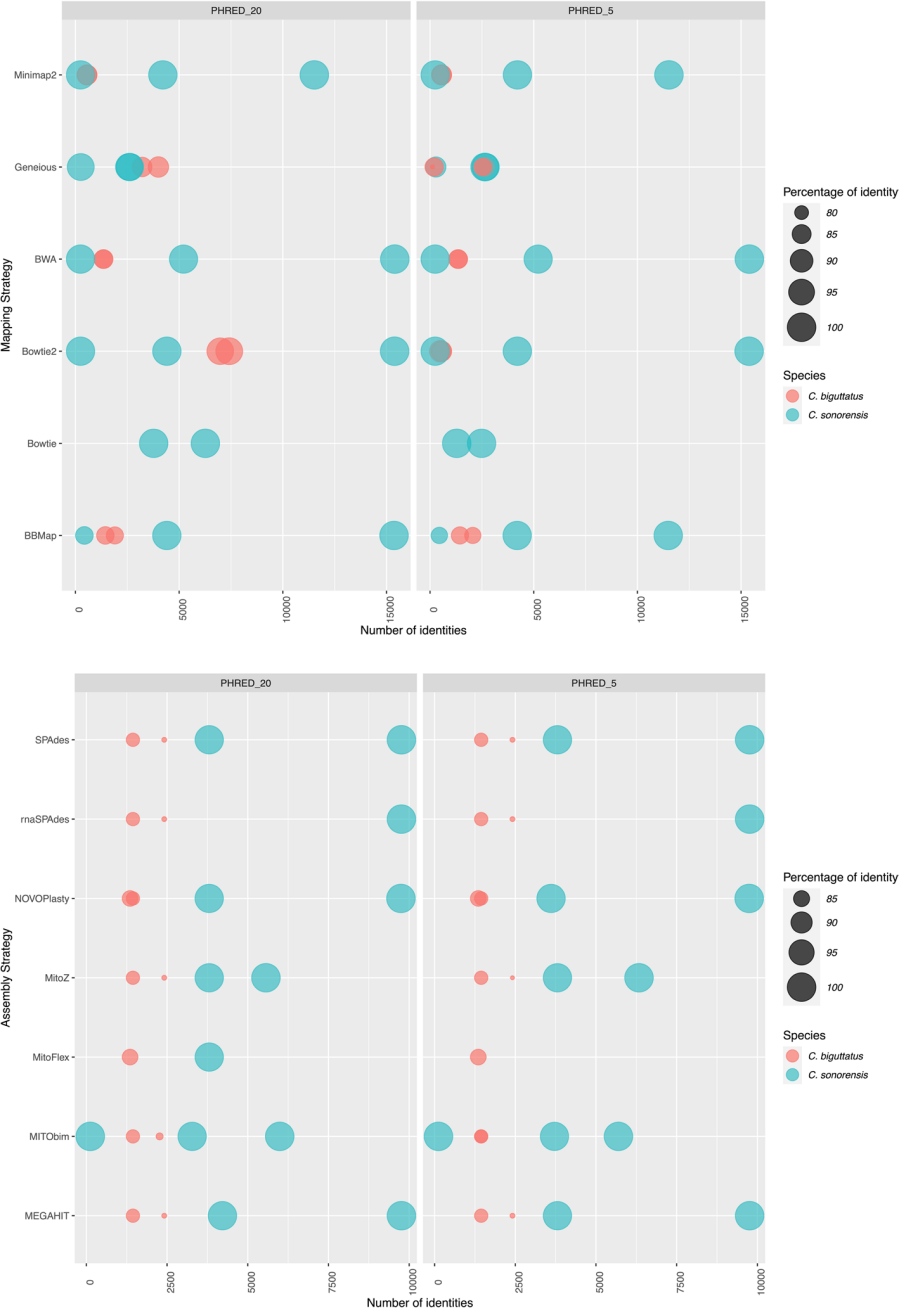
Comparison of the performance of the two strategies for mitogenome generation. **A.** Mapping strategy, including the six mappers BWA v0.7.17 [15, 16], Bowtie2 v2.4.4 [17], Bowtie v1.3.1 [18], Minimap2 v2.17 [19], BBMap v38.84 [20], and Geneious [Biomatters Ltd.]. **B.** De novo assembly strategy using the seven assemblers MEGAHIT v1.2.9 [18], SPAdes v3.14.1 [19], rnaSPAdes v3.14.1 [20], MitoFlex v0.2.9 [21], MitoZ v2.3 [22], MITObim v 1.9.1 [23], and NOVOPlasty v2.7.2 [3]

BWA produced the best results for *C. sonorensis* (Fig. 3) for both filtering approaches (PHRED 5 and 20), generating consensus sequences of 15,404 bp (*C. sonorensis*_F004 [PHRED 20]) and 15,407 bp (*C. sonorensis*_F002 [PHRED 20]) (Fig. 3). Those consensus sequences resulted in 15,398 and 5,214 identical sites (IS) and in 99.97% and 99.33% pairwise identity (PI) compared to the reference (scaffold710, GenBank accession number LN484060.1 [30]), respectively (Fig. 3). Bowtie2 was the second-best mapper with 99.97% PI, 15,391 IS (*C. sonorensis*_F004) and 99.64% PGI, 4,409 IS (*C. sonorensis*_F002), respectively. The consensus sequences derived from the Geneious mapper were among the lowest in percentages of pairwise genetic identity and identical sites for *C. sonorensis* (Fig. 3).

For *C. biguttatus*, Bowtie2 resulted in the highest number of identical sites and percentage of pairwise identity to the most closely related non-specific reference mitochondrial genome available (*Culicoides arakawae* AB361004.1) (Fig. 3). More specifically, 7,418 IS and 96.74% PI was recovered for *C. biguttatus*_G02 when filtering at PHRED 20, and 6,984 IS and 96.3% PI for *C. biguttatus*_G04 at PHRED 20. Geneious ranked second, with 3,217 IS, and 85.73% PI for *C. biguttatus*_G04 (PHRED 20) and 4,002 IS and 86.89% PI for *C. biguttatus*_G02 (PHRED 20), followed by BBMap (2,062 IS and 82.11% PI-*C. biguttatus*_G04) and BWA (1,354 IS and 84.73% PI-*C. biguttatus*_G04), while Bowtie did not generate consensus sequences or good alignments in any of the tests.

Despite Geneious ranking second in terms of IS, the mapper consistently recovered the highest number of differences for *C. biguttatus* mitogenomes when compared to the reference mitochondrial genome, doubling the number of differences seen in the second-best mapper (e.g. for *C. biguttatus*_G04, 983 [Geneious] vs 371 [Bowtie2] nucleotide differences). For *C. biguttatus*, the Bowtie2 mapper was able to generate a consensus sequence of 18,140 bp and 18,137 bp for *C. biguttatus*_G04 (PHRED 20) and *C. biguttatus*_G02 (PHRED 5), respectively. Geneious generated consensus sequences of 18,708 bp for *C. biguttatus*_G04 (PHRED 20) and 18,687 bp for *C. biguttatus*_G02 (PHRED 20). However, when using the *C. sonorensis* reference mitogenome instead of the *C. arakawae* mitogenome for reconstructing the mitogenome of *C. biguttatus* with Bowtie2, the resulting mitogenome of *C. biguttatus* was more similar to that of *C. sonorensis* than that of *C. arakawae*, which indicated a high dependency on the used reference genome in absence of a species-specific reference (Supplementary Figure S2). Due to this strong mapping bias, we selected a *de novo* assembled consensus sequence for *C. biguttatus* (PHRED 20) to represent the mitogenome generated for the species.

### Evaluation of mitogenomes based on de novo assembly

All *de novo* assemblers failed to recover complete mitogenomes for specimens that underwent mitochondrial isolation prior to DNA extraction, likely due to the extremely low number of reads per specimen, which was further reduced after trimming and quality filtering (Supplementary Table S1). MEGAHIT, rnaSPAdes, and SPAdes ranked highest in terms of IS (9,761) and percentage of PI (100%) across the assembled sequences for *C. sonorensis*_F004, regardless of the filtering strategy (PHRED 5 and 20) when comparing their assembled sequences to the reference scaffold710 (GenBank accession number LN484060.1 [30] (Fig. 3). However, sequence lengths for *C. sonorensis*_F004 varied by filtering strategy, being identical (13,613 bp) when filtering at PHRED 20 and varying when filtering at PHRED 5 (MEGAHIT: 13,619 bp, SPAdes: 13, 918 bp, rnaSPAdes: 13,619 bp). MEGAHIT performed the best for *C. sonorensis*_F002 (PHRED 20) with 4,216 IS, and 99.74% PI (Fig. 3) over 15,539 bp, and MITObim recovered the lowest metrics (3,278 IS and 99.7% PI [Fig. 3]) over 10,203 bp at PHRED 20.

For *C. biguttatus_*G04, MEGAHIT (PHRED 5 and 20), SPAdes (PHRED 5 and 20), rnaSPAdes (PHRED 5 and 20), and MitoZ (PHRED 20) performed the best with 2,413 IS and 80.26% of PI (Fig. 3). The longest assembled mitogenome for *C. biguttatus*_G04 was 14,417 bp long (SPAdes). For *C. biguttatus_*G02, NOVOPlasty, MitoZ, MITObim, MEGAHIT, SPAdes, and rnaSPAdes ranked highest with identical metrics (1,443 IS and 83.12% PI) [Fig. 3]. The longest assembled mitogenome for *C. biguttatus*_G02 was 8,556 bp long (MEGAHIT).

### *Culicoides* mitogenome description

**Fig. 4:**
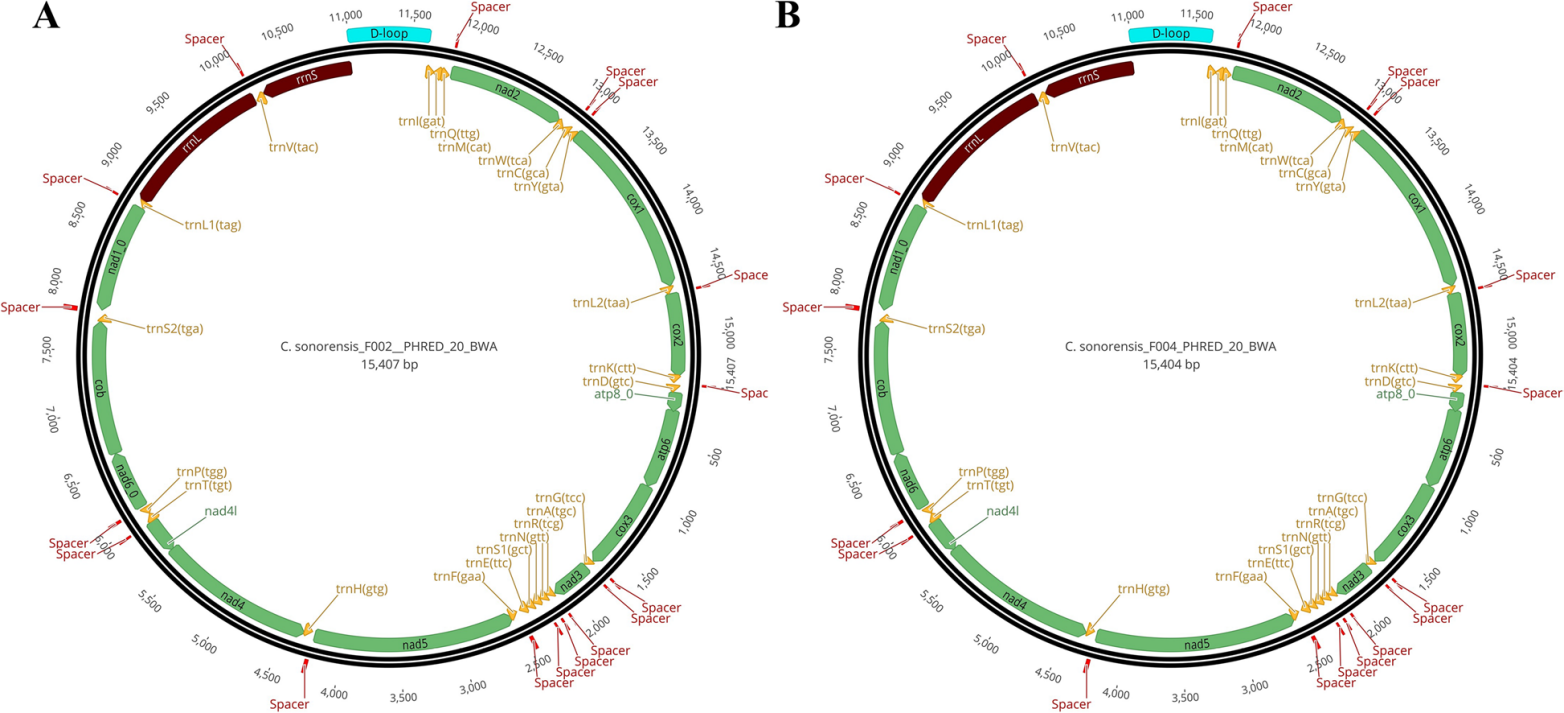
Mitochondrial genome organization of *C. sonorensis*_F002 (**A**), and *C. sonorensis*_F004 (**B**). PCGs, rRNA, and tRNA are indicated in green, brown, and orange. The control region (D-loop) is indicated in blue, and all other intergenic spacers are indicated in red

Mitogenome size for the species ranged from 15,404 to 15,407 bp in *C. sonorensis*_F004 (GenBank accession number ON758299) and *C. sonorensis_*F002 (GenBank accession number ON758298), respectively (Fig. 4). GC content for both haplotypes was identical at 21.5%. Near-complete mitogenomes were reconstructed from the mapping strategy with only four unidentified nucleotides (Ns) in *C. sonorensis_*F004 and one N in *C. sonorensis*_F002. The Ns were within the non-coding region (D-loop) for *C. sonorensis*_F004 and within the rRNA S for *C. sonorensis*_F002). Consequently, all 37 coding genes were successfully recovered and annotated for *C. sonorensis* (Fig. 4) with at least one annotator(e.g., MITOS2). For *C. sonorensis*_F004 and *C. sonorensis*_F002, PCG sizes ranged from 159 (ATP8) to 1,722 bp (NAD5), while tRNA and rRNA sizes varied from 61 [tRNA C(gca)] to 72 bp [tRNA V(tac)] and 783 (rRNA S) to 1,313 (rRNA L), respectively. The inferred sizes of the control region were 637 bp for *C. sonorensis*_F004 and 638 bp *C. sonorensis*_F002. The start codon for all protein-coding regions was the standard ATN (coding for I) start codon except for COI, which used CGA (coding for R). No variation was seen in the stop codons of protein-coding regions, which was inferred as TAA in all cases, although some showed an incomplete signal (e.g., only T) [Supplementary Table S2].

**Fig. 5:**
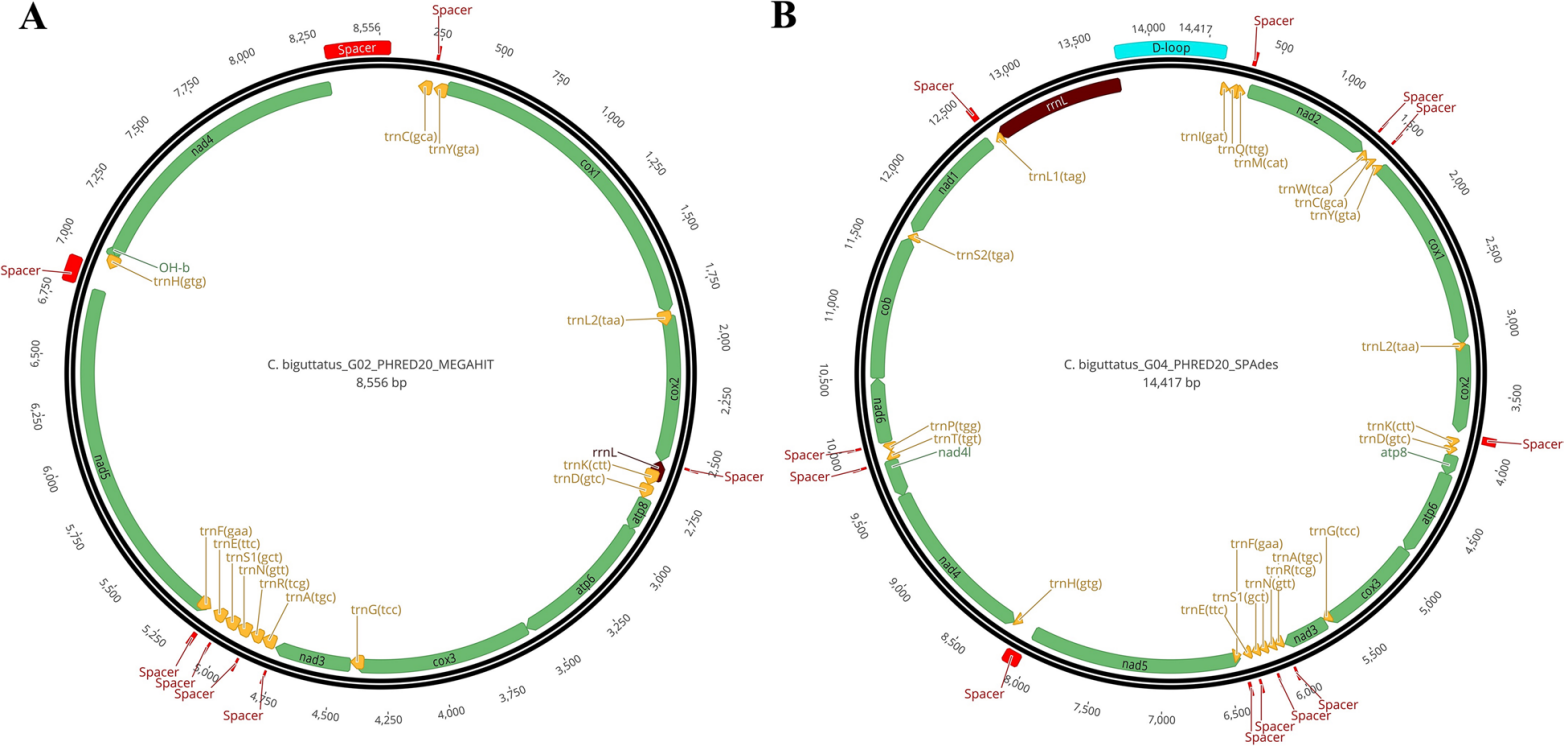
Mitochondrial genome organization of *Culicoides biguttatus*. *C. biguttatus*_G02 (**A**) and *C. biguttatus*_G04 (**B**). PCGs, rRNA, and tRNA are indicated in green, brown, and orange. The control region (D-loop) is indicated in blue, and all other intergenic spacers are indicated in red

Only partial mitochondrial genomes were generated for *C. biguttatus* (Fig. 5). The most complete mitogenome generated by de novo assembling presented 14,417 bp (8,556 bp for *C. biguttatus*_G02 [PHRED 20], GenBank accession number ON758300) and 26.7% of GC content. The *C. biguttatus*_G04 mitogenome (GenBank accession number ON758301) exhibited a GC content equal to 27.6% (Fig. 5). Thirty-five out of 37 genes in the mitogenome of *C. biguttatus*_G04 were recovered, with only rRNA SSU and tRNA V gene missing. In *C. biguttatus*_G02, 14 genes were not found (trnI, trnM, trnS2, trnL1, trnQ, trnP, trnT, trnV, trnW, rrnS, nad1, nad2, nad6, and cob) when compared to *Culicoides arakawae* mitogenome [accession number AB361004.1] and *Culicoides sonorensis* (Fig. 4 A) [accession number LN484060.1]. In the case of *C. biguttatus*, PCG sizes ranged from 159 (ATP8) to 1,665 bp (NAD5). tRNA and rRNA sizes varied from 60 [tRNA R(tcg)] to 71 bp [tRNA K(ctt)] in both haplotypes. SSU rRNA was missing in both haplotypes. On the contrary, LSU rRNA had a size of 1,042 (rRNA L) in *C. biguttatus*_G04 and a truncated and transposed fragment was only found for *C. biguttatus*_G02 [rRNA L (92 bp)]. The inferred size of the control region was 794 bp for *C. biguttatus*_G04. Similar to *C. sonorensis*, the start codon for all protein-coding regions was the standard ATN (coding for I) start codon except for COI, which used CCG in this case (coding for P). All protein-coding genes showed identical stop codons inferred as TAA, although some exhibited an incomplete signal (e.g., only T) [Supplementary Table S2].

### Comparison of the best mitogenomes generated in each approach

The best mitogenome for *C. sonorensis* (*C. sonorensis*_F004) based on the mapping strategy with BWA had a higher percentage of identity (99.97) and nucleotide identities (15,398) than that based on the de novo assembly strategy with any tool. The best mitogenome was generated for *C. biguttatus*_G04 when using MEGAHIT, rnaSPAdes, SPAdes, and MitoZ, which all scored equal (2,413 IS and 80.26% PI). For *C. biguttatus*_G02, the mitogenome based on MEGAHIT ranked highest among the assembling tools (1,443 IS and 83.12% PI, [Fig. 3]). Overall, the de novo assembling strategy generated a higher quality and non-biased mitogenome for all specimens.

Both applied quality filtering approaches (PHRED scores 5 and 20) allowed the generation of high-quality mitogenomes, confirming sufficient quality of the raw reads for mitogenome generation. More stringent quality trimming at PHRED 20 resulted in the best results for both the mapper and de novo assembly strategies for *C. sonorensis* and *C. biguttatus* specimens in most cases. The best mitogenomes for both species generated with the most stringent quality filtering (PHRED 20) were annotated and used as the most complete, newly described mitogenomes for *Culicoides*.

Overall gene arrangement was identical among *C. sonorensis*, *C. biguttatus* and *C. arakawae*. However, several intergenic fragments of different sizes were observed across *Culicoides* mitogenomes, mainly responsible for differences in mitochondrial genome sizes. A total of 17 spacers were found in *C. sonorensis*, ranging from 1 to 30 bp. On the other hand, *C. biguttatus*' most complete mitogenome showed up to 12 spacers ranging from 2 to 114 bp. Altogether, spacers occupied 298 bp in *C. biguttatus*_G04. The largest intergenic spacer in *C. biguttatus* was found between the genes tRNA H and NAD5 (114 bp). In *C. sonorensis* the largest spacer had 30 bp and were found between rRNA E and tRNA F and between tRNA S2 and NAD1. Inferred control region sizes ranged from 638 bp in *C. sonorensis* to 794 bp in *C. biguttatus*.

**Fig. 6:**
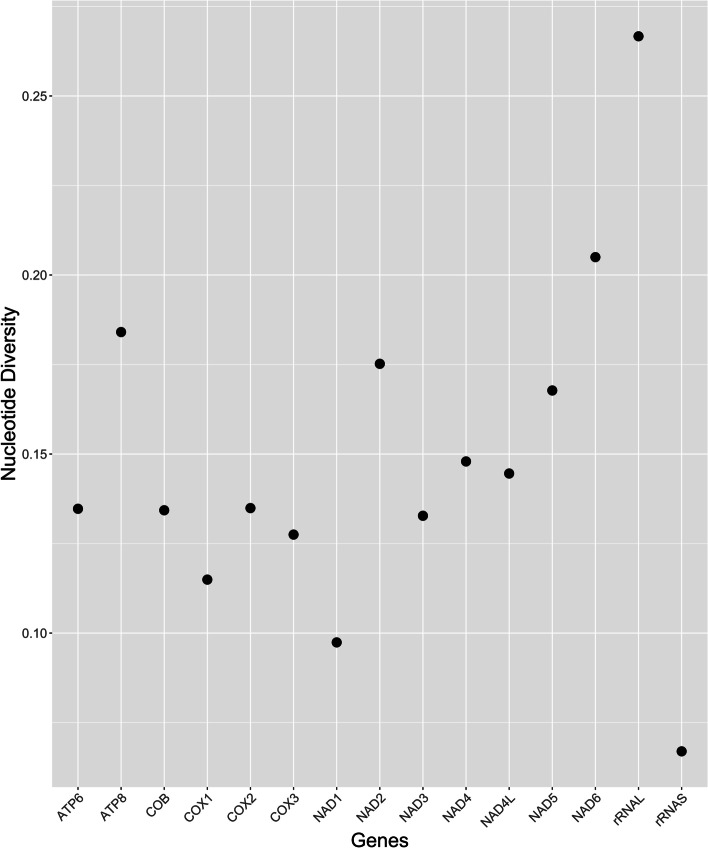
Nucleotide diversity per mitochondrial protein-coding and rRNA genes within *Culicoides* as inferred from all mitogenomes available, including the haplotypes generated in the present study

Each generated mitogenome was evaluated using the reference sequence to determine the pairwise genetic identity and identical sites. When aligning the mitogenome sequences generated for both haplotypes within species, they were over 99% identical within *C. sonorensis* and *C. biguttatus*. The latter shows a highly similar haplotype composition. An analysis by gene revealed that genes ATP8, NAD2, NAD6, and LSU rRNA showed the highest nucleotide diversity (Fig. 6) and pairwise interspecific genetic distance (Supplementary Figure S3), with COI presenting the third-lowest metrics among all mitochondrial genes.

## Discussion

Mitogenome analysis provides a unique insight into genome and taxa evolution and sets the basis for successfully detecting high-resolution mitochondrial markers for species identification and, particularly, the use of the whole mitogenome as a marker [31]. All eight specimens used here were morphologically and molecularly identified to the species level to avoid taxonomic uncertainty when reporting their associated mitogenome information. Average nucleotide diversity among *Culicoides* mitochondrial genes suggests that the ATP8, NAD2, NAD6, LSU rRNA genes are the most variable mitochondrial markers, followed by the NAD4, NAD4L, and NAD5 genes. This confirms previous observations that NADH genes evolve faster than cytochrome oxidase genes [9]. The latter was the case for *Culicoides* mitogenomes except for NAD1. In accordance with the higher nucleotide substitution rate, ATP8, NAD2, NAD6, and LSU rRNA genes also exhibited the highest average pairwise genetic distances among *Culicoides*' available mitogenomes. Patterns of genetic variation among these genes suggest they could be suitable gene combinations for establishing an effective DNA-based identification system for *Culicoides*. Some of these genes, such as LSU rRNA, have proven their utility in identifying and reconstructing the phylogeny of insect species of forensic importance [31]. ATP8 and NAD6 have also shown higher genetic variation than COI in other insects (e.g., Lepidopterans), which has suggested a disproportionate focus on COI within this group for the species identification [32]. Therefore, mitochondrial gene variation analysis within the order Lepidoptera has revealed COI among the least variable genes in the species mitogenomes and confirmed other genes with higher genetic variability, such as NAD2, NAD4, and NAD5 [33]. An ideal DNA barcode marker should have a rate of molecular evolution high enough to provide maximum discrimination among the compared species. No compelling a priori reason supports the focus on COI over any other mitochondrial protein-coding gene [4]. However, proper mitochondrial molecular markers selection for other *Culicoides* species identification or other derived applications of mitogenome information cannot be established without the characterization of their mitogenome and the establishment of comprehensive DNA reference databases. Currently, there are 414 (out of 1,347) *Culicoides* species with available COI records on BOLD (http://www.boldsystems.org/) as of May 2^nd^, 2022. Molecular information is missing for approximately 70% of the recognized species in the genus. Given the limited data, it is not straightforward to demonstrate that the standard mitochondrial barcode marker (COI) works as expected for all *Culicoides* or that this marker is outperformed by the other potential mitochondrial markers. Enough empirical information suggests COI is an insufficient molecular marker for species identification in some Diptera families [34]. In the *Culicoides* context, COI showed the third-lowest values of nucleotide diversity among *Culicoides* mitogenomes under study. However, the complete COI gene reconstructed in the present study from the MiSeq reads unambiguously validated the specimens’ identity and the identity of their associated mitogenome. Despite the sufficiency of the complete COI gene to provide identification for the *Culicoides* species under study, it is important to highlight that these species represent less than 1% of the total *Culicoides*, and further evaluation of understudied species will be needed to check if this pattern holds.

In addition, standard DNA-barcoding of the specimens using the COI molecular marker might be biased in the presence of exogenous DNA material within the blood meal (e.g., host DNA). Consequently, additional efforts to validate the identity of the generated mitochondrial genomes are needed [9]. The mitogenome of the host *Bos taurus* was recovered from *C. biguttatus* DNA reads, confirming the potential of metagenomics for studying vectors and their hosts simultaneously, as well as potentially vectored pathogens [35–37]. Notably, all *C. biguttatus* specimens used for mitogenome reconstruction were sampled in a dairy barn.

In the present study, no mitogenome was generated when isolating the mitochondria before whole mtDNA extraction due to the low mtDNA concentrations and number of reads associated with mtDNA. The low mtDNA concentrations are inevitably linked to the limited amount of starting material, which presents a challenge regardless of the applied mitochondrial isolation methods/kits and can only be increased by pooling multiple individuals prior to DNA extraction. Pooling individuals was not considered in the present experimental design to avoid further complications during mitogenome reconstruction, given the possibility of the presence of different haplotypes and since the mix of genetic variants is a recognized challenge during insect genome assembly and annotation [36]. The low number of generated mtDNA reads might also be due to the lower depth of coverage used when sequencing these libraries (up to tenfold less than that for whole-genome sequencing libraries), which should be increased in future attempts. Nevertheless, only eight insect mitogenomes have successfully been sequenced based on mitochondrial isolation [38]. Consequently, the vast majority of insect mitogenomes sequenced to date (up to 98%) have been generated without mitochondrial isolation [33], which reaffirms the limitations of the isolation procedure (e.g., laborious protocols and additional costs per sample). Direct shotgun sequencing for mitogenome sequencing also has the key advantage that the approach requires less amount of tissue, which was essential for the present study, and is faster than approaches that involve mitochondrial isolation [33]. Consequently, given the success in generating full mitogenomes for *Culicoides* species from single specimens without mitochondrial isolation, future mitogenome sequencing efforts could skip mitochondrial isolation to avoid extra protocol steps and costs per sample processing. If mitochondrial isolation is desired, improving mtDNA isolation efficiency or using whole-genome amplification kits after isolation should be explored to increase mtDNA concentrations. Furthermore, a significantly higher depth of coverage than that used in this study will be needed to validate mitochondrial isolation as a practical approach for mitogenome sequencing in *Culicoides*.

### Mapping to a reference mitogenome versus de novo assembly

Mapping to a reference mitogenome was demonstrated to be a more accurate approach than de novo assembly to generate *Culicoides* mitochondrial genomes in the presence of a species-specific reference mitogenome. However, mapping results were biased in the absence of a species-specific reference genome. In the latter case, *de novo* assemblers were deemed more reliable. Our comparison of *de novo* assemblers included both metagenomics- and metatranscriptomics-optimized assemblers to compare their performances for mitogenome assemblies. Since both optimization types were among the best performing *de novo* assemblers, the assembly performance seems to be assembler-dependent rather than optimization-dependent. Overall, BWA can be considered an efficient, fast, and accurate mapper for *Culicoides* mitogenome generation using MiSeq Illumina reads in the presence of species-specific reference mitogenomes. Mapping strategies are, in general, faster, and computationally less demanding than *de novo* assembly and might become essential as the number of available mitochondrial reference genomes increases. However, this approach is not free of limitations, and checking for potential mapping bias will be necessary when more reference-quality *Culicoides* mitochondrial genomes are being released. To date, only one *Culicoides* reference mitogenome is available in NCBI Reference Sequence Database. In the absence of specific reference mitogenomes, three main *de novo* assembling tools, MEGAHIT, SPAdes, and MitoZ, should be considered for optimal *Culicoides* mitogenome reconstruction. The most complete mitogenomes were generated for the specimens *C. sonorensis*_F004 and *C. biguttatus*_G04 using mapping and *de novo* assembling approaches, respectively.

Haplotype comparison within *C. sonorensis* and *C. biguttatus* revealed over 99% sequence similarity, respectively, confirming the accuracy of the best mapping and assembling tools. Overall, gene arrangement and organization of *C. sonorensis and C. biguttatus* mitogenomes were identical to the one reported for *Drosophila yakuba* (GenBank accession number NC_001322.1 /[11]), which represents the ancestral gene organization of insect mitogenomes [11]. Consequently, no translocation of tRNA was observed in the generated mitogenomes, as has been reported for other *Culicoides* species [29]. GC content was 21.5% and 26.7% for *C. sonorensis* and *C. biguttatus*, respectively, exhibiting the extreme AT bias observed in insect mitogenomes [10, 32]. A long intergenic segment of 114 bp was found in the genome region of *C. biguttatus* between tRNA H and NAD5 genes. Long non-coding regions (from 65 to 1,846 pb) have been described in the mitogenome of other six *Culicoides* species, including *C. arakawae* [29]. Those long non-coding segments might contain high molecular variation, although there might be functional constraints if it becomes demonstrated that they play a role in mitogenome replication and transcription. This hypothesis is based on the fact that the long spacers are AT-rich segments similar to the control region and prone to form secondary structures (e.g. stem-loops) [29]. The presence of common intergenic spacers for the same *Culicoides* mitogenome region (e.g., between tRNA H and NAD5 genes) in divergent species like *C. biguttatus* and *C. sonorensis* (present study) and *C. arakawae* [29]) might also be indicative of the common insertion origin of the segment during evolution. Potential explanations to the origin of these intergenic spacers are duplication, translocation from the control region, DNA polymerase slippage during replication, or even horizontal transfer from nuclear DNA [29]. However, further analysis will be necessary to determine the function and origin of these spacers.

## Conclusions and future direction

We provide two novel annotated mitogenomes for *Culicoides*, which might function as a baseline for unravelling the poorly resolved phylogenetic relationships among *Culicoides* species and establishing additional mitochondrial markers for molecular *Culicoides* identification. We propose that the ATP8, NAD2, NAD6, and LSU rRNA genes could be potential barcodes for *Culicoides*. Future studies should focus on expanding both the available intraspecific and interspecific mitogenome information for the genus *Culicoides* in order to confirm the applicability of these genes and determine the best delineation of interspecific and intraspecific genetic distances. Whether or not COI remains sufficient for the remaining *Culicoides* species or is outperformed by other mitochondrial genes will require further evaluation as more *Culicoides* mitogenomes become available.

Our methodology might be useful for similar studies on *Culicoides*. We recommended the mapping tool BWA for mitogenome reconstruction in the presence of species-specific reference mitogenome and the annotation tool MITOS2 for mitogenome annotation. *De novo* assemblers SPAdes, MEGAHIT, and MitoZ are recommended in the lack of a species-specific reference genome for *Culicoides* to avoid mapping bias. We plan to sequence the mitogenomes of other *Culicoides* species, including the 38 *Culicoides* species currently described for Ontario, and eventually reach the 57 species found in Canada [36, 39]. The establishment of a complete molecular database containing all genomes of all viral serotypes transmitted by *Culicoides* will be beneficial for the detection of viral DNA in *Culicoides* HTS datasets. Similarly, the establishment of a complete genome database containing all *Culicoides* hosts will be beneficial for rapid screening during the molecular identification of biting midges. Landscape alteration and climate change are recognized causes of *Culicoides´* geographic expansions beyond their native ranges [39]. Molecular tools to allow their identification are critical to adequately assessing health risk, implementing effective management plans, or simply evaluating undocumented diversity due to geographic range shifts. *Culicoides* species have a broad range of hosts and represent severe health and economic concerns, from wildlife and livestock health to tourism, highlighting the importance of molecular biomonitoring and biosurveillance [39]. Therefore, establishing such databases might allow the quick molecular identification of vectored pathogens, vectors, and their hosts to develop effective mitigation plans and avoid massive economic loss.

## Methods

### Specimen selection

**Fig. 7:**
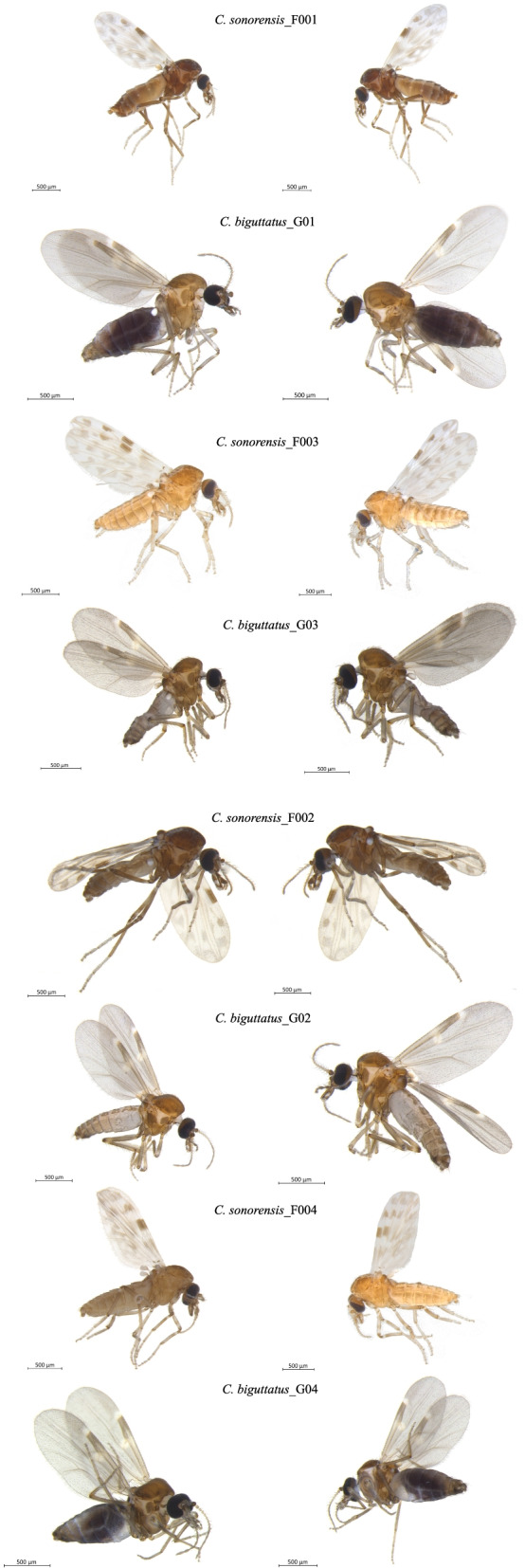
Lateral images (left and right) of the *Culicoides* specimens used in the present study. The dark macula in the abdomen of *C. biguttaus*_G01 indicates blood meal. All specimens in this figure are female. Images were organized by treatment received, where all the specimens listed here underwent mitochondrial isolation previous mitogenome sequencing. Lateral images (left and right) of the *Culicoides* specimens used in the present study. The dark macula in the abdomen of *C. biguttaus*_G04 indicates blood meal. All specimens in this figure are female. Images were organized by treatment received, where all the specimens listed here underwent direct mitogenome sequencing without mitochondrial isolation.

Two *Culicoides* species were chosen for the present study, *Culicoides sonorensis* and *C. biguttatus* (Fig. 7). All *C. biguttatus* specimens were collected with miniature Centre for Disease Control (CDC) light traps with UV lights (Bioquip, CA, USA) at the Ontario Veterinary College Dairy Barn, University of Guelph, Guelph, ON Canada [36]. *C. sonorensis* specimens belonged to a colony of specimens that originated in Idaho (Owyhee County). The colony has been maintained since 1973 by the United States Department of Agriculture/Agricultural Research Service, Arthropod-Borne Animal Disease Research Unit (USDA/ARS, ABADRU) in Manhattan, KS. These *C. sonoresis* specimens used in our study were acquired in 2016 by the Canadian Food Inspection Agency (CFIA) at the National Centre for Animal Diseases, Lethbridge Laboratory. All specimens were imaged before DNA extraction using a Leica MC170 HD camera mounted on a Leica M205 A microscope utilizing the software Leica LAS X (version 3.6.0.20104; Leica Microsystems, Wetzlar, Germany), (Fig. 7). Taxonomic classifications of the collected *Culicoides* specimens to species level were based on morphological key characters [40–44].

Four specimens per species were subjected to two different treatments: treatment one consisted of mitochondrial isolation followed by genomic extraction (*C. sonorensis*_F001 /- CFIALET2016-COLAK-F001, *C. sonorensis*_F003 /- CFIALET2016-COLAK-F003, *C. biguttatus_*G01 / FAP3ST-DAB02-025, and *C. biguttatus*_G03 / FAP3ST-DAB02-027), and treatment two consisted of whole genomic DNA extraction (*C. sonorensis*_F002 / CFIALET2016-COLAK-F002, *C. sonorensis*_F004 / CFIALET2016-COLAK-F004, *C. biguttatus*_G02 / FAP3ST-DAB02-026, and *C. biguttatus*_G04 / FAP3ST-DAB02-028). Mitochondrial isolation was conducted with the Qproteome Mitochondria Isolation Kit (Qiagen catalogue # 37,612) following the manufacturer's instructions.

### DNA extraction

Genomic DNA extractions were performed with the MagAttract High Molecular Weight (HMW) DNA extraction kit (Qiagen, Toronto, ON Canada) using mitochondrial fractions and whole specimens. For the mitochondrial fractions, the kit was used as indicated by the manufacturer. Modifications to the manufacturer’s recommendations were necessary when extracting from the whole specimen and are outlined as follows: Whole *Culicoides* specimens were weighed on an analytical scale (Sartorius, Oakville, ON Canada). Each specimen’s weight was < 1 mg, which was lower than the 25 mg of tissue weight recommended in the DNA extraction kit handbook. Despite the insufficient tissue weight of each specimen, individual specimens were processed instead of pooling different specimens from the same species; to avoid complications in the analysis due to the presence of multiple haplotypes. A total of 220 µL of ATL buffer and 20 µL of proteinase K were added to each specimen, and the specimens were incubated overnight at 56ºC with shaking at 700 rpm to ensure complete digestion. The lysates were subsequently transferred to 2 mL centrifuge tubes containing approximately 250 mg of 1 mm glass beads. The lysate of each specimen was disrupted in a TissueLyser II (Qiagen, Toronto, ON Canada) at a frequency of 30 Hz for one minute and then incubated for additional two hours at 56ºC with shaking at 700 rpm, following the addition of another 20 µL volume of proteinase K. After the second incubation, any remaining visible tissue was physically disrupted using a sterile pestle before proceeding with the protocol. This thorough chemical and physical tissue degradation was chosen to achieve the maximum DNA yield possible for the very low tissue amount of starting material per specimen. Once specimens were lysed entirely, the manufacturer DNA extraction protocol for fresh/frozen tissue was followed. DNA extracts were run on a 1% agarose gel and quantified by fluorometry using a Qubit and the dsDNA HS kit (Thermo Fisher Scientific, Mississauga, ON Canada) with 10 µL of each extract. DNA quantities were measured three times per sample (every ten minutes) and average concentrations and standard deviations were calculated (Fig. 1). The integrity of DNA extracts was further validated by amplifying the COI barcode gene with the COI-specific primers and PCR conditions described in Milián-García et al. 2020 and 2021 [36, 45] during the first steps of library preparation for Illumina MiSeq sequencing.

### Illumina Sequencing

High-Throughput Sequencing was conducted using an Illumina MiSeq System and a MiSeq reagent kit version 3 (600 cycles) at the Advance Analysis Center (AAC), University of Guelph. The samples were prepared for sequencing using the Nextera XT library prep kit (FC-131–1096) from Illumina and Nextera XT v2 indices (FC-131-200X) according to Illumina’s instructions with one modification: Macherey–Nagel NucleoMag NGS Beads (REF 744,970) were used instead of AMPure XP beads, which does not affect protocol performance and considerably reduces sample processing costs. Insert sizes were 400 bp for whole specimen samples and 50–200 bp for mitochondrial fraction samples. DNA libraries were pooled so that each sample from whole specimen DNA received 10% of the total MiSeq reads and each sample from mitochondrial fractions received 1% of the total reads. The difference in sequence depths per sample type was based on the assumption that 1% out of a conservative total of 17 × 10^6^ reads generated in a MiSeq run would be enough to sequence a mitochondrial genome of 20 kbp at 2,550X depth of coverage. More sequencing effort was needed to sequence the mitochondrial genome when using whole specimen DNA, as it contains nuclear DNA as well, which is why samples from whole specimen DNA received an order of magnitude more reads. All sequencing reads were demultiplexed, and the Illumina adapters were trimmed using the MiSeq Reporter software, generating two paired-end FastQ raw data files. Read quality was checked using FastQC (http://www.bioinformatics.babraham.ac.uk/projects/fastqc/), and FastQC reports of all specimens were summarized using MultiQC [46] for better visualization. We received 7,813,084 300 bp paired-end reads in total, and specimens that underwent mitochondrial isolation prior to DNA extraction received 4,872–12,624 reads, which was one to three orders of magnitudes less reads than that received by specimens that underwent whole genomic DNA extraction (Supplementary Table S1).

### Trimming and mitogenome generation based on mapping to reference genomes

For every sample, forward and reverse reads were trimmed, and quality filtered using BBDuk v38.84. Quality filtering was performed at two different PHRED scores (5 and 20) to explore the effect of stringent and relaxed quality filtering on mitogenome generation. The PHRED scores allowed for a 25% and 1% chance of error during base calling, respectively. Trimming included removing all Truseq, Nextera, and PhiX adapters if present. All other BBDuk parameters were kept at default, including discarding of reads shorter than 10 bp. The trimmed and quality-filtered reads were then mapped against a reference mitogenome.

Mitogenome generation for each specimen based on mapping to reference genomes was conducted using the six mappers BWA v0.7.17-r1198-dirty with the BWA-MEM algorithm [15, 16], Bowtie2 v2.4.4 [17], Bowtie v1.3.1 [18], Minimap2 v2.17 [19], BBMap v38.84 [20], and Geneious (https://www.geneious.com) as implemented in Geneious Prime v2021.2.2. For *C. sonorensis* samples, the sequence “scaffold710” from GenBank (accession number LN484060.1) [30] and for *C. biguttatus* samples, the validated mitochondrial genome sequence for *C. arakawae* available in the RefSeq database (accession number AB361004.1/ NC_009809.1), were used as reference sequences [29]. The settings of all mappers were kept at default except for the mapping sensitivity, which was set to “highest” in all but the Geneious mapper (“high”) to reduce the runtime, and BWA, for which no specific mapping sensitivity option was available. Consensus sequences that resulted from mapping were manually checked, and sequence terminals with missing data at the 5’ and 3’ terminals were deleted. Only outputs that resulted in five or more reads mapped against the reference sequence were considered, as five non-overlapping reads of 300 bp length are sufficient to cover the entire COI gene (approximately 1,500 bp). The COI gene is considered the standard molecular barcode for metazoans [4] and was the only barcode gene that we amplified after the DNA extractions and prior to sequencing to evaluate the DNA integrity (Fig. 1). All consensus sequences generated from the mappers were aligned against the reference mitogenome using LASTZ v1.02.00 [47, 48] as a genome alignment tool implemented in Geneious and keeping the parameters at default. LASTZ allowed the estimation of the pairwise number of identical sites (IS) and the percentage of pairwise identity (%PI) among compared sequences. In cases of multiple alignment arrangements, only the combination with the highest IS and %PI was considered for further analysis. The consensus sequence of the mapper with the best performance, that is, the highest numbers of identical sites (IS) and percentage of pairwise identity (%PI) that at the same time minimize the number of differences to the reference sequence was selected as the best-generated mitogenome sequence. The identical sites metric does not consider ambiguities. In contrast, the percentage of pairwise identity considers them up to 50% identical to the compared position if the ambiguity includes the nucleotide present on the reference. Both IS and %PI limit the comparison between the commonly sequenced and overlapping DNA regions. Differences in non-overlapping sequences were used as a third metric to take this variation into account when choosing the best consensus sequence. Using the mapper yielding the best performance, the trimmed and quality-filtered reads of *C. biguttatus* specimens with blood meal (*C. biguttatus_*G01 and *C. biguttatus_*G04) were mapped against potential host mitogenome (*Bos taurus*, GenBank accession number NC_006853).

Furthermore, potential mapping bias when using different reference mitogenomes was evaluated for *C. biguttatus*, as it was the only species lacking a species-specific reference. In this case, mapping against both available reference mitogenomes (*C. arakawae* and *C. sonorensis*) was completed for *C. biguttatus* specimens G02 and G04, using the mapper yielding the best results for *C. biguttatus*. The generated mitogenomes using both references were used for a haplotype comparison in a phylogenetic tree in the context of all available *Culicoides* mitogenomes. We aligned sequences using MAFFT v 7.450 [49] with default parameters as implemented in Geneious (https://www.geneious.com). Bayesian phylogenetic analysis was completed using MrBayes v3.2.6 [47] and *Aedes aegypti* as an outgroup (GenBank accession number NC_035159.1), a GTR substitution model, and gamma-distributed rate variation. Four simultaneous chains with a chain length of 1,000,000 were run, each using a random tree as a starting point, a subsampling frequency of 200, and the default heating scheme. As burn-in samples, the first 2,000 trees were discarded. The remaining trees were used to derive posterior probability values and construct a majority-rule consensus tree. The expectation was that in the absence of mapping bias, the relationship of the haplotypes would remain the same regardless of the reference mitogenome used. On the contrary, if the mapping to a non-specific reference mitogenome was biasing the output, the haplotype would shift the relationship to being more closely related to the reference.

### Mitogenome generation based on de novo assembly

Forward and reverse reads were trimmed, and quality filtered as described above. To investigate the impact of different assemblers on mitogenome assembly, trimmed and quality-filtered reads were assembled using five *de novo* assemblers, including MEGAHIT v1.2.9 [21], SPAdes v3.14.1 [22], rnaSPAdes v3.14.1 [23], the MitoFlex v0.2.9 [24] assembly module, and the MitoZ v2.3 [25] assembly module, and two seed-based assemblers, including MITObim v 1.9.1 [26] and NOVOPlasty v2.7.2 [3]. MitoFlex and MitoZ are modular pipelines specifically designed for mitogenome assembly and annotation. The MitoFlex and MitoZ assembly modules represent a modified version of the assemblers MEGAHIT and SOAPdenovo-Trans [50], respectively, to better assemble mitochondrial sequences. MEGAHIT, SPAdes, and the MEGAHIT-modified MitoFlex assembly module are metagenomics-optimized *de novo* assemblers, whereas rnaSPAdes and the SOAPdenovo-Trans-modified MitoZ assembly module are metatranscriptomics-optimized *de novo* assemblers. Since mitochondrial reads have much higher copy numbers than other reads, metagenomics- and metatranscriptomics-optimized assemblers were tested to compare their performances for mitogenome assemblies.

MEGAHIT, SPAdes, and rnaSPAdes were run with default parameters. For the MitoZ assembly module, it was specified that the reads stemmed from a specimen in the clade Arthropoda, read length was 300 bp and insert size was 400 bp. The assemblies were run in quick mode, which involved only one k-mer length, limited the assembly to sequences belonging to the clade Arthropoda, and all other default parameters were used. For the MitoFlex assembly module, the parameter to specify insert length as 400 bp was specified and all other default parameters were used.

The two seed-based assemblers required a seed as a starting point of the assembly. For *C. biguttatus* assemblies, the COI reference sequence of BIN ID BOLD:AAG6468 from the Barcode of Life Data System (BOLD) database was used as a seed. In lack of a comparably reliable COI reference sequence for *C. sonorensis*, “Culicoides sonorensis COI” was searched in the NCBI GenBank database (https://www.ncbi.nlm.nih.gov/genbank/), all available *C. sonorensis* COI sequences were downloaded and imported into Geneious. The sequences were aligned using MAFFT v7.450 [51], with the consensus threshold set to 50%, to minimize ambiguous bases in the consensus sequence. The exported consensus sequence was used as a seed. MITObim was run for 100 iterations and all other default parameters were used. NOVOPlasty was run with default parameters as given in the configuration file example on GitHub (https://github.com/ndierckx/NOVOPlasty).

To select the appropriate mitochondrial genome sequence for further analysis among all assembled sequences of each assembler, the MitoZ findmitoscaf module was initially used, which was designed to identify mitochondrial sequences among assembled sequences and involved profile Hidden Markov Models, protein-coding gene (PCG) annotation, and NUMTs and contamination removal. However, the MitoZ findmitoscaf module failed on all assembly results for both *C. sonorensis* and *C. biguttatus* and stated an error that the read coverage in the forward and reverse reads used for assembly was too low (“All sequences are low abundance (< 10X)”). This indicated that MitoZ was not able to verify potential mitochondrial sequences due to too low read abundances. As an alternative, we generated a BLAST database using only the validated mitochondrial genome sequence for *C. arakawae* available in the RefSeq database (accession number AB361004.1/ NC_009809.1) and BLASTed all assembled sequences against the *C. arakawae* BLAST database. If a sequence had any match with the database, it was considered a mitochondrial sequence. For each assembler, we selected the longest matched sequence as the longest generated mitochondrial genome sequence for further analysis. Among these selected sequences, the best *de novo* mitogenome sequence was selected as described for the mapping strategy using the same reference sequence per species.

### Mitogenome annotations

To investigate the impact of different annotation tools on mitogenome annotations, the best mitogenome sequence based on the mapping and *de novo* approach, respectively, was annotated using four different annotation tools, including two web-server-based tools (MITOS2 [52] and GeSeq [53]) and the two command-line-based annotation modules of MitoZ [54] and MitoFlex [55]. Furthermore, all reference mitogenomes used in this study were annotated de novo or re-annotated with MITOS2, GeSeq, MitoZ, and MitoFlex, using the same parameters as in the analyses indicated above, to check the accuracy in reproducing the same results of the already annotated mitogenomes (e.g. for *C. arakawae* [29] *C. sonorensis* Scaffold710, and *D. yakuba* [11]). For MITOS2, a web server for the annotation of metazoan and fungi mitochondrial genomes, “RefSeq 89 Metazoa'' as a reference, “5 Invertebrates” as the genetic code, and all other default settings were used. Visualization and mitogenome illustration of all annotations were conducted in Geneious. Furthermore, MITOS2-annotated COI genes were extracted to validate the specimens’ identity based on COI barcodes using the top BLAST hit against the NCBI GenBank database and the identification engine from BOLD.

For GeSeq, a web server for rapid and accurate organelle genome annotation, “mitochondrial” was selected as the sequence source and ARWEN v1.2.3 [56] as the 3^rd^ party tRNA annotator with search mode “Metazoan Mitochondrial tRNAs” and genetic code “Invertebrate Mitochondrial.” The taxon chosen from the NCBI RefSeq database to annotate against, that was the only available taxon of the genus *Culicoides*, was *C. arakawae* (NC_009809.1). MitoZ and MitoFlex annotation modules were run for the clade Arthropoda and all other default parameters were used. The different annotation tools were compared based on the number of genes identified and general gene arrangement in generated mitogenomes. Only the best annotation tool output was chosen for *Culicoides* mitogenome representation. The specific gene arrangement that resulted in the best annotations was compared among the sequenced haplotypes and the reference mitogenomes using CREx [57]. Genome annotations were visualized, checked, and all intergenic segments (spacers) were manually annotated in Geneious (https://www.geneious.com). Annotated genes for each available *Culicoides* haplotype (*C. sonorensis*_F02, *sonorensis*_F04, *C. biguttatus*_G02, *C. biguttatus*_G04, *C. sonorensis* LN484060.1, and *C. arakawae* NC_009809.1) were independently aligned using MAFFT v 7.450 [49] with default parameters as implemented in Geneious (https://www.geneious.com). Each alignment was then used to estimate nucleotide diversity and pairwise p*-*genetic distances with pegas [58] and ape [59] R packages. Posterior data visualization and figure preparation were completed in R v4.0.2 [60] with ggplot2 [61] and plotly [62] packages.

## Supplementary Information

**Additional file 1: Table S1**. Extraction methods and the number of 300 bp paired-end reads per specimen.

**Additional file 2: Supplementary Table S2.** MITOS2 annotations obtained for each mitogenome generated in the present study, including gene names, start and stop positions, strand, length of the genes in terms of nucleotides, and start/stop codons for the protein-coding genes (PCGs).

**Additional file 3: Supplementary Figure S1.** *Bos taurus* mitogenome recovered from *Culicoides biguttatus*. A. Reference mitogenome for *Bos taurus* (NC_006853) and B. *Bos taurus* mitogenome generated from *C. biguttatus*_G04 containing blood meal. PCGs, rRNA, and tRNA are indicated in green, brown, and orange. The control region (D-loop) is noted in blue.

**Additional file 4: Supplementary Figure S2.** Bayesian reconstruction of the relationship among *Culicoides* mitogenomes generated up to date, including current haplotypes. A. Blue square indicates *C. biguttatus* haplotypes mapped against *C. arakawae*. B. The red square indicates *C. biguttatus* haplotypes mapped against *C. sonorensis*_Scaffold710 using the same mapper (Bowtie2). Notice the switch in position of *C. biguttatus* depending on the reference mitogenome used. The numbers over the nodes indicate posterior probability support. *Aedes aegypti* (GenBank accession number NC_035159.1) represents the outgroup.

**Additional file 5: Supplementary Figure S3.** Heat map representing pairwise p genetic distances among *Culicoides* mitogenomes haplotypes available up to date.

## Data Availability

The datasets generated (Raw data [FASTQ files]) and/or analyzed during the current study are available in the University of Guelph Research Data Repository (https://dataverse.scholarsportal.info/dataset.xhtml?persistentId=doi:10.5683/SP3/UQIIXN) and GenBank (Accession numbers ON758298, ON758299, ON758300, and ON758301). Specimen images are available from the Barcode of Life Data System (DS-CUMG2021; dx.doi.org/10.5883/DS-CUMG2021).

## References

[1] Lavrov DV, Pett W (2016). Animal Mitochondrial DNA as We Do Not Know It: mt-Genome Organization and Evolution in Nonbilaterian Lineages. Genome Biol Evol.

[2] DeSalle R, Hadrys H (2017). Evolutionary Biology and Mitochondrial Genomics: 50 000 Mitochondrial DNA Genomes and Counting. eLS. American Cancer Society.

[3] Dierckxsens N, Mardulyn P, Smits G (2017). NOVOPlasty: De novo assembly of organelle genomes from whole genome data. Nucleic Acids Res.

[4] Hebert PDN, Cywinska A, Ball SL, deWaard JR (2003). Biological identifications through DNA barcodes. Proc Biol Sci.

[5] Galtier N, Nabholz B, Glémin S, Hurst GDD (2009). Mitochondrial DNA as a marker of molecular diversity: a reappraisal. Mol Ecol.

[6] Boore JL (1999). Animal mitochondrial genomes. Nucleic Acids Res.

[7] Das J (2006). The role of mitochondrial respiration in physiological and evolutionary adaptation. BioEssays.

[8] Ye F, Li H, Xie Q (2021). Mitochondrial Genomes from Two Specialized Subfamilies of Reduviidae (Insecta: Hemiptera) Reveal Novel Gene Rearrangements of True Bugs. Genes.

[9] Dong Z, Wang Y, Li C, Li L, Men X (2021). Mitochondrial DNA as a Molecular Marker in Insect Ecology: Current Status and Future Prospects. Ann Entomol Soc Am.

[10] Cameron SL (2014). Insect Mitochondrial Genomics: Implications for Evolution and Phylogeny. Annu Rev Entomol.

[11] Clary DO, Wolstenholme DR (1985). The mitochondrial DNA molecule of Drosophila yakuba: nucleotide sequence, gene organization, and genetic code. J Mol Evol.

[12] De Bruijn LMH (1983). Drosophila melanogaster mitochondrial DNA, a novel organization and genetic code. Nature.

[13] Shao R, Campbell NJH, Barker SC (2001). Numerous Gene Rearrangements in the Mitochondrial Genome of the Wallaby Louse, Heterodoxus macropus (Phthiraptera). Mol Biol Evol.

[14] Formenti G, Rhie A, Balacco J, Haase B, Mountcastle J, Fedrigo O (2021). Complete vertebrate mitogenomes reveal widespread repeats and gene duplications. Genome Biol.

[15] Li H, Durbin R (2009). Fast and accurate short read alignment with Burrows-Wheeler transform. Bioinformatics.

[16] Li H, Durbin R (2010). Fast and accurate long-read alignment with Burrows-Wheeler transform. Bioinformatics.

[17] Langmead B, Salzberg SL (2012). Fast gapped-read alignment with Bowtie 2. Nat Methods.

[18] Langmead B, Trapnell C, Pop M, Salzberg SL (2009). Ultrafast and memory-efficient alignment of short DNA sequences to the human genome. Genome Biol.

[19] Li H (2018). Minimap2: pairwise alignment for nucleotide sequences. Bioinformatics.

[20] Bushnell B (2014). BBMap: A Fast, Accurate, Splice-Aware Aligner.

[21] Li D, Liu CM, Luo R, Sadakane K, Lam TW (2015). MEGAHIT: An ultra-fast single-node solution for large and complex metagenomics assembly via succinct de Bruijn graph. Bioinformatics.

[22] Bankevich A, Nurk S, Antipov D, Gurevich AA, Dvorkin M, Kulikov AS (2012). SPAdes: A New Genome Assembly Algorithm and Its Applications to Single-Cell Sequencing. J Comput Biol.

[23] Bushmanova E, Antipov D, Lapidus A, Prjibelski AD (2019). rnaSPAdes: a de novo transcriptome assembler and its application to RNA-Seq data. GigaScience.

[24] Li JY, Li WX, Wang AT, Zhang Y (2021). MitoFlex: an efficient, high-performance toolkit for animal mitogenome assembly, annotation and visualization. Bioinformatics.

[25] Meng G, Li Y, Yang C, Liu S (2019). MitoZ: a toolkit for animal mitochondrial genome assembly, annotation and visualization. Nucleic Acids Res.

[26] Hahn C, Bachmann L, Chevreux B (2013). Reconstructing mitochondrial genomes directly from genomic next-generation sequencing reads - A baiting and iterative mapping approach. Nucleic Acids Res.

[27] Mellor PS, Boorman J, Baylis M (2000). Culicoides biting midges: their role as arbovirus vectors. Annu Rev Entomol.

[28] Borkent A, Dominiak P (2020). Catalog of the Biting Midges of the World (Diptera: Ceratopogonidae). Zootaxa.

[29] Matsumoto Y, Yanase T, Tsuda T, Noda H (2009). Species-specific mitochondrial gene rearrangements in biting midges and vector species identification. Med Vet Entomol.

[30] Morales-Hojas R, Hinsley M, Armean IM, Silk R, Harrup LE, Gonzalez-Uriarte A (2018). The genome of the biting midge Culicoides sonorensis and gene expression analyses of vector competence for bluetongue virus. BMC Genomics.

[31] Stevens J, Wall R (2001). Genetic relationships between blowflies (Calliphoridae) of forensic importance. Forensic Sci Int.

[32] Wu FF, Gao Q, Liu F, Wang Z, Wang JL, Wang XG (2020). DNA barcoding evaluation of Vicia (Fabaceae): Comparative efficacy of six universal barcode loci on abundant species. J Syst Evol.

[33] Cameron SL, Whiting MF (2008). The complete mitochondrial genome of the tobacco hornworm, Manduca sexta, (Insecta: Lepidoptera: Sphingidae), and an examination of mitochondrial gene variability within butterflies and moths. Gene.

[34] Nelson LA, Lambkin CL, Batterham P, Wallman JF, Dowton M, Whiting MF (2012). Beyond barcoding: a mitochondrial genomics approach to molecular phylogenetics and diagnostics of blowflies (Diptera: Calliphoridae). Gene.

[35] Borland EM, Kading RC (2021). Modernizing the Toolkit for Arthropod Bloodmeal Identification. Insects.

[36] Milián-García Y, Janke LAA, Young RG, Ambagala A, Hanner RH (2021). Validation of an Effective Protocol for Culicoides Latreille (Diptera: Ceratopogonidae) Detection Using eDNA Metabarcoding. Insects.

[37] Konstantinidis K, Bampali M, de Courcy Williams M, Dovrolis N, Gatzidou E, Papazilakis P (2022). Dissecting the Species-Specific Virome in Culicoides of Thrace. Front Microbiol.

[38] Cameron SL (2014). How to sequence and annotate insect mitochondrial genomes for systematic and comparative genomics research. Syst Entomol.

[39] Allen SE, Vigil SL, Jardine CM, Furukawa-Stoffer T, Colucci N, Ambagala A, et al. New Distribution Records of Biting Midges of the Genus Culicoides (Diptera: Ceratopogonidae) Latreille, Culicoides bergi and Culicoides baueri, in Southern Ontario, Canada. J Med Entomol. 2022;tjac047:1467–72. 10.1093/jme/tjac047 35468207

[40] Jamnback H, Wirth WW (1963). The Species of *Culicoides* Related to obsoletus in Eastern North America (Diptera: Ceratopogonidae). Ann Entomol Soc Am.

[41] Foote RH, Pratt HD (1954). The *Culicoides* of the Eastern United States (Diptera, Heleidae), a Review.

[42] Hoffman WA (1925). A Review of the Species of *Culicoides* of North and Central America and the West Indies. Am J Hyg..

[43] Wirth WW, Dyce AL, Peterson BV, Roper I (1984). An atlas of wing photographs, with a summary of the numerical characters of the Nearctic species of *Culicoides* (Diptera: Ceratopogonidae). Contrib Am Entomol Inst..

[44] Root FM, Hoffman WA (1937). The North American species of *Culicoides*. Am J Epidemiol.

[45] Milián-García Y, Young RG, Madden M, Bullas-Appleton E, Hanner RH (2020). Optimization and validation of a cost-effective protocol for biosurveillance of invasive alien species. Ecol Evol.

[46] Ewels P, Magnusson M, Lundin S, Käller M (2016). MultiQC: summarize analysis results for multiple tools and samples in a single report. Bioinformatics.

[47] Harris RS (2007). Improved pairwise alignment of genomic DNA. Ph.D.

[48] Schwartz S (2003). Human-Mouse Alignments with BLASTZ. Genome Res.

[49] Katoh K, Standley DM (2013). MAFFT Multiple Sequence Alignment Software Version 7: Improvements in Performance and Usability. Mol Biol Evol.

[50] Xie Y, Wu G, Tang J, Luo R, Patterson J, Liu S (2014). SOAPdenovo-Trans: De novo transcriptome assembly with short RNA-Seq reads. Bioinformatics.

[51] Katoh K, Standley DM (2013). MAFFT Multiple Sequence Alignment Software Version 7: Improvements in Performance and Usability Article Fast Track. Mol Biol Evol..

[52] Donath A, Jühling F, Al-Arab M, Bernhart SH, Reinhardt F, Stadler PF (2019). Improved annotation of protein-coding genes boundaries in metazoan mitochondrial genomes. Nucleic Acids Res.

[53] Tillich M, Lehwark P, Pellizzer T, Ulbricht-Jones ES, Fischer A, Bock R (2017). GeSeq - Versatile and accurate annotation of organelle genomes. Nucleic Acids Res.

[54] Meng G, Li Y, Yang C, Liu S (2019). MitoZ: a toolkit for animal mitochondrial genome assembly, annotation and visualization. Nucleic Acids Res.

[55] Li JY, Li WX, Wang AT, Zhang Y. MitoFlex: an efficient, high-performance toolkit for animal mitogenome assembly, annotation and visualization. Bioinformatics. 2021;btab111:3001–3. 10.1093/bioinformatics/btab111 33605414

[56] Laslett D, Canbäck B (2008). ARWEN: a program to detect tRNA genes in metazoan mitochondrial nucleotide sequences. Bioinformatics.

[57] Bernt M, Merkle D, Ramsch K, Fritzsch G, Perseke M, Bernhard D (2007). CREx: inferring genomic rearrangements based on common intervals. Bioinformatics.

[58] Paradis E (2010). pegas: an R package for population genetics with an integrated–modular approach. Bioinformatics.

[59] Paradis E, Schliep K (2019). ape 5.0: an environment for modern phylogenetics and evolutionary analyses in R. Bioinformatics.

[60] R Core Team (2020). A language and environment for statistical computing.

[61] Wickham H. ggplot2: elegant graphics for data analysis. Switzerland: Springer; 2016.

[62] Sievert C. Interactive web-based data visualization with R, plotly, and shiny. Boca Raton: CRC Press; 2020.

