# Supplementary material for: Mitochondrial genome sequencing, mapping, and assembly benchmarking for Culicoides species (Diptera: Ceratopogonidae)
Source: BMC Genomics. 2022 Aug 13;23:584. doi: 10.1186/s12864-022-08743-x (PMC9375341; doi:10.1186/s12864-022-08743-x)
Supplement: Supplementary file 1 — Additional file 1: Table S1. Extraction methods and the number of 300 bp paired-end reads per specimen. [file 12864_2022_8743_MOESM1_ESM.docx]

Table S1. Extraction methods and the number of 300 bp paired-end reads per specimen.

| **Specimen** | **Extraction method** | **# of reads** |
| --- | --- | --- |
| *C. biguttatus*_G01 | Mitochondrial isolation | 4,872 |
| *C. biguttatus*_G02 | Whole genomic DNA | 2,521,946 |
| *C. biguttatus*_G03 | Mitochondrial isolation | 9,100 |
| *C. biguttatus*_G04 | Whole genomic DNA | 2,294,368 |
| *C. sonorensis*_F001 | Mitochondrial isolation | 21,356 |
| *C. sonorensis*_F002 | Whole genomic DNA | 2,248,700 |
| *C. sonorensis*_F003 | Mitochondrial isolation | 12,624 |
| *C. sonorensis*_F004 | Whole genomic DNA | 700,118 |
