# Supplementary material for: Mitochondrial genome sequencing, mapping, and assembly benchmarking for Culicoides species (Diptera: Ceratopogonidae)
Source: BMC Genomics. 2022 Aug 13;23:584. doi: 10.1186/s12864-022-08743-x (PMC9375341; doi:10.1186/s12864-022-08743-x)
Supplement: Supplementary file 2 — Additional file 2: Supplementary Table S2. MITOS2 annotations obtained for each mitogenome generated in the present study, including gene names, start and stop positions, strand, length of the genes in terms of nucleotides, and start/stop codons for the protein-coding genes (PCGs). [file 12864_2022_8743_MOESM2_ESM.docx]

Supplementary Table S2. MITOS2 annotations obtained for each mitogenome generated in the present study, including gene names, start and stop positions, strand, length of the genes in terms of nucleotides, and start/stop codons for the protein-coding genes (PCGs).

*C. sonorensis*_F002_PHRED 20_BWA

| **Name** | **Start** | **Stop** | **Strand** | **Length** | **Start/Stop codons** |
| --- | --- | --- | --- | --- | --- |
| trnC(gca) | 12911 | 12971 | - | 61 |  |
| trnR(tcg) | 2181 | 2242 | + | 62 |  |
| trnL1(tag) | 8778 | 8839 | - | 62 |  |
| trnP(tgg) | 6026 | 6089 | - | 64 |  |
| trnL2(taa) | 14572 | 14635 | + | 64 |  |
| trnS2(tga) | 7748 | 7812 | + | 65 |  |
| trnH(gtg) | 4266 | 4330 | - | 65 |  |
| trnG(tcc) | 1694 | 1758 | + | 65 |  |
| trnF(gaa) | 2472 | 2536 | - | 65 |  |
| trnE(ttc) | 2377 | 2441 | + | 65 |  |
| trnA(tgc) | 2115 | 2179 | + | 65 |  |
| trnY(gta) | 12974 | 13039 | - | 66 |  |
| trnT(tgt) | 5960 | 6025 | + | 66 |  |
| trnS1(gct) | 2311 | 2376 | + | 66 |  |
| trnN(gtt) | 2245 | 2310 | + | 66 |  |
| trnI(gat) | 11635 | 11700 | + | 66 |  |
| trnW(tca) | 12852 | 12918 | + | 67 |  |
| trnD(gtc) | 5 | 71 | + | 67 |  |
| trnQ(ttg) | 11698 | 11766 | - | 69 |  |
| trnM(cat) | 11764 | 11833 | + | 70 |  |
| trnK(ctt) | 15328 | 15398 | + | 71 |  |
| trnV(tac) | 10143 | 10214 | - | 72 |  |
| atp8 | 72 | 230 | + | 159 | ATC/TAA |
| nad4l | 5661 | 5957 | - | 297 | ATG/TAA |
| nad3 | 1765 | 2112 | + | 348 | ATA/TAA |
| nad6 | 6110 | 6613 | + | 504 | ATT/TAA |
| atp6 | 224 | 901 | + | 678 | ATG/TAA |
| cox2 | 14640 | 15347 | + | 708 | ATG/TAA |
| rrnS | 10214 | 10996 | - | 783 |  |
| cox3 | 901 | 1689 | + | 789 | ATG/TAA |
| nad1 | 7843 | 8776 | - | 934 | ATG/T(AA) |
| nad2 | 11846 | 12853 | + | 1008 | ATT/TAA |
| cob | 6613 | 7749 | + | 1137 | ATG/TAA |
| rrnL | 8817 | 10129 | - | 1313 |  |
| nad4 | 4330 | 5667 | - | 1338 | ATG/TAA |
| cox1 | 13041 | 14576 | + | 1536 | CGA/TAA |
| nad5 | 2520 | 4241 | - | 1722 | ATT/TAA |

*C. sonorensis*_F004_PHRED_20_BWA

| **Name** | **Start** | **Stop** | **Strand** | **Length** | **Start/Stop codons** |
| --- | --- | --- | --- | --- | --- |
| trnC(gca) | 12908 | 12968 | - | 61 |  |
| trnR(tcg) | 2181 | 2242 | + | 62 |  |
| trnL1(tag) | 8776 | 8837 | - | 62 |  |
| trnP(tgg) | 6024 | 6087 | - | 64 |  |
| trnL2(taa) | 14569 | 14632 | + | 64 |  |
| trnE(ttc) | 2377 | 2440 | + | 64 |  |
| trnT(tgt) | 5959 | 6023 | + | 65 |  |
| trnS2(tga) | 7746 | 7810 | + | 65 |  |
| trnH(gtg) | 4265 | 4329 | - | 65 |  |
| trnG(tcc) | 1694 | 1758 | + | 65 |  |
| trnF(gaa) | 2471 | 2535 | - | 65 |  |
| trnA(tgc) | 2115 | 2179 | + | 65 |  |
| trnY(gta) | 12971 | 13036 | - | 66 |  |
| trnS1(gct) | 2311 | 2376 | + | 66 |  |
| trnN(gtt) | 2245 | 2310 | + | 66 |  |
| trnI(gat) | 11632 | 11697 | + | 66 |  |
| trnW(tca) | 12849 | 12915 | + | 67 |  |
| trnD(gtc) | 5 | 71 | + | 67 |  |
| trnQ(ttg) | 11695 | 11763 | - | 69 |  |
| trnM(cat) | 11761 | 11830 | + | 70 |  |
| trnK(ctt) | 15325 | 15395 | + | 71 |  |
| trnV(tac) | 10141 | 10212 | - | 72 |  |
| atp8 | 72 | 230 | + | 159 | ATC/TAA |
| nad4l | 5660 | 5956 | - | 297 | ATG/TAA |
| nad3 | 1765 | 2112 | + | 348 | ATA/TAA |
| nad6 | 6108 | 6611 | + | 504 | ATT/TAA |
| atp6 | 224 | 901 | + | 678 | ATG/TAA |
| cox2 | 14637 | 15344 | + | 708 | ATG/TAA |
| rrnS | 10212 | 10994 | - | 783 |  |
| cox3 | 901 | 1689 | + | 789 | ATG/TAA |
| nad1 | 7841 | 8774 | - | 934 | ATG/T(AA) |
| nad2 | 11843 | 12850 | + | 1008 | ATT/TAA |
| cob | 6611 | 7747 | + | 1137 | ATG/TAA |
| rrnL | 8815 | 10127 | - | 1313 |  |
| nad4 | 4329 | 5666 | - | 1338 | ATG/TAA |
| cox1 | 13038 | 14573 | + | 1536 | CGA/TAA |
| nad5 | 2519 | 4240 | - | 1722 | ATT/TAA |

*C. biguttatus*_G02_PHRED 20

| **Name** | **Start** | **Stop** | **Strand** | **Length** | **Start/Stop codons** |
| --- | --- | --- | --- | --- | --- |
| trnR(tcg) | 4852 | 4911 | + | 60 |  |
| trnC(gca) | 181 | 241 | - | 61 |  |
| trnH(gtg) | 6924 | 6986 | - | 63 |  |
| trnE(ttc) | 5050 | 5112 | + | 63 |  |
| trnY(gta) | 253 | 316 | - | 64 |  |
| trnL2(taa) | 1849 | 1912 | + | 64 |  |
| trnF(gaa) | 5131 | 5194 | - | 64 |  |
| trnG(tcc) | 4358 | 4422 | + | 65 |  |
| trnS1(gct) | 4981 | 5046 | + | 66 |  |
| trnN(gtt) | 4915 | 4980 | + | 66 |  |
| trnA(tgc) | 4787 | 4852 | + | 66 |  |
| trnD(gtc) | 2675 | 2741 | + | 67 |  |
| trnK(ctt) | 2604 | 2674 | + | 71 |  |
| rrnL | 2555 | 2646 | - | 92 |  |
| atp8 | 2742 | 2900 | + | 159 | ATC/TAA |
| nad4l | 8317 | 45 | - | 285 | ATG/TAA |
| nad3 | 4423 | 4776 | + | 354 | ATA/TAA |
| atp6 | 2894 | 3571 | + | 678 | ATG/TAA |
| cox2 | 1871 | 2552 | + | 682 | ATG/T(AA) |
| cox3 | 3571 | 4398 | + | 828 | ATG/TAA |
| nad4 | 6986 | 8323 | - | 1338 | ATG/TAA |
| cox1 | 315 | 1853 | + | 1539 | CCG/TAA |
| nad5 | 5145 | 6809 | - | 1665 | ATT/TAA |

*C. biguttatus*_G04_PHRED 20

| **Name** | **Start** | **Stop** | **Strand** | **Length** | **Start/Stop codons** |
| --- | --- | --- | --- | --- | --- |
| trnR(tcg) | 6096 | 6155 | + | 60 |  |
| trnC(gca) | 1425 | 1485 | - | 61 |  |
| trnL1(tag) | 12673 | 12734 | - | 62 |  |
| trnH(gtg) | 8168 | 8229 | - | 62 |  |
| trnI(gat) | 131 | 193 | + | 63 |  |
| trnE(ttc) | 6294 | 6356 | + | 63 |  |
| trnY(gta) | 1497 | 1560 | - | 64 |  |
| trnT(tgt) | 9859 | 9922 | + | 64 |  |
| trnS2(tga) | 11643 | 11706 | + | 64 |  |
| trnL2(taa) | 3093 | 3156 | + | 64 |  |
| trnF(gaa) | 6375 | 6438 | - | 64 |  |
| trnP(tgg) | 9923 | 9987 | - | 65 |  |
| trnG(tcc) | 5602 | 5666 | + | 65 |  |
| trnS1(gct) | 6225 | 6290 | + | 66 |  |
| trnN(gtt) | 6159 | 6224 | + | 66 |  |
| trnA(tgc) | 6031 | 6096 | + | 66 |  |
| trnW(tca) | 1366 | 1432 | + | 67 |  |
| trnD(gtc) | 3919 | 3985 | + | 67 |  |
| trnQ(ttg) | 191 | 259 | - | 69 |  |
| trnM(cat) | 257 | 325 | + | 69 |  |
| trnK(ctt) | 3848 | 3918 | + | 71 |  |
| atp8 | 3986 | 4144 | + | 159 | ATC/TAA |
| nad4l | 9560 | 9856 | - | 297 | ATG/TAA |
| nad3 | 5667 | 6020 | + | 354 | ATA/TAA |
| nad6 | 10002 | 10508 | + | 507 | ATT/TAA |
| atp6 | 4138 | 4815 | + | 678 | ATG/TAA |
| cox2 | 3115 | 3796 | + | 682 | ATG/T(AA) |
| cox3 | 4815 | 5642 | + | 828 | ATG/TAA |
| nad1 | 11697 | 12629 | - | 933 | ATT/TAA |
| nad2 | 350 | 1360 | + | 1011 | ATT/TAA |
| rrnL | 12712 | 13753 | - | 1042 |  |
| cob | 10508 | 11644 | + | 1137 | ATG/TAA |
| nad4 | 8229 | 9566 | - | 1338 | ATG/TAA |
| cox1 | 1559 | 3097 | + | 1539 | CCG/TAA |
| nad5 | 6389 | 8053 | - | 1665 | ATT/TAA |
