# Supplementary material for: Mitochondrial genome sequencing, mapping, and assembly benchmarking for Culicoides species (Diptera: Ceratopogonidae)
Source: BMC Genomics. 2022 Aug 13;23:584. doi: 10.1186/s12864-022-08743-x (PMC9375341; doi:10.1186/s12864-022-08743-x)
Supplement: Supplementary file 3 — Additional file 3: Supplementary Figure S1. Bos taurus mitogenome recovered from Culicoides biguttatus. A. Reference mitogenome for Bos taurus (NC_006853) and B. Bos taurus mitogenome generated from C. biguttatus_G04 containing blood meal. PCGs, rRNA, and tRNA are indicated in green, brown, and orange. The control region (D-loop) is noted in blue. [file 12864_2022_8743_MOESM3_ESM.pdf]

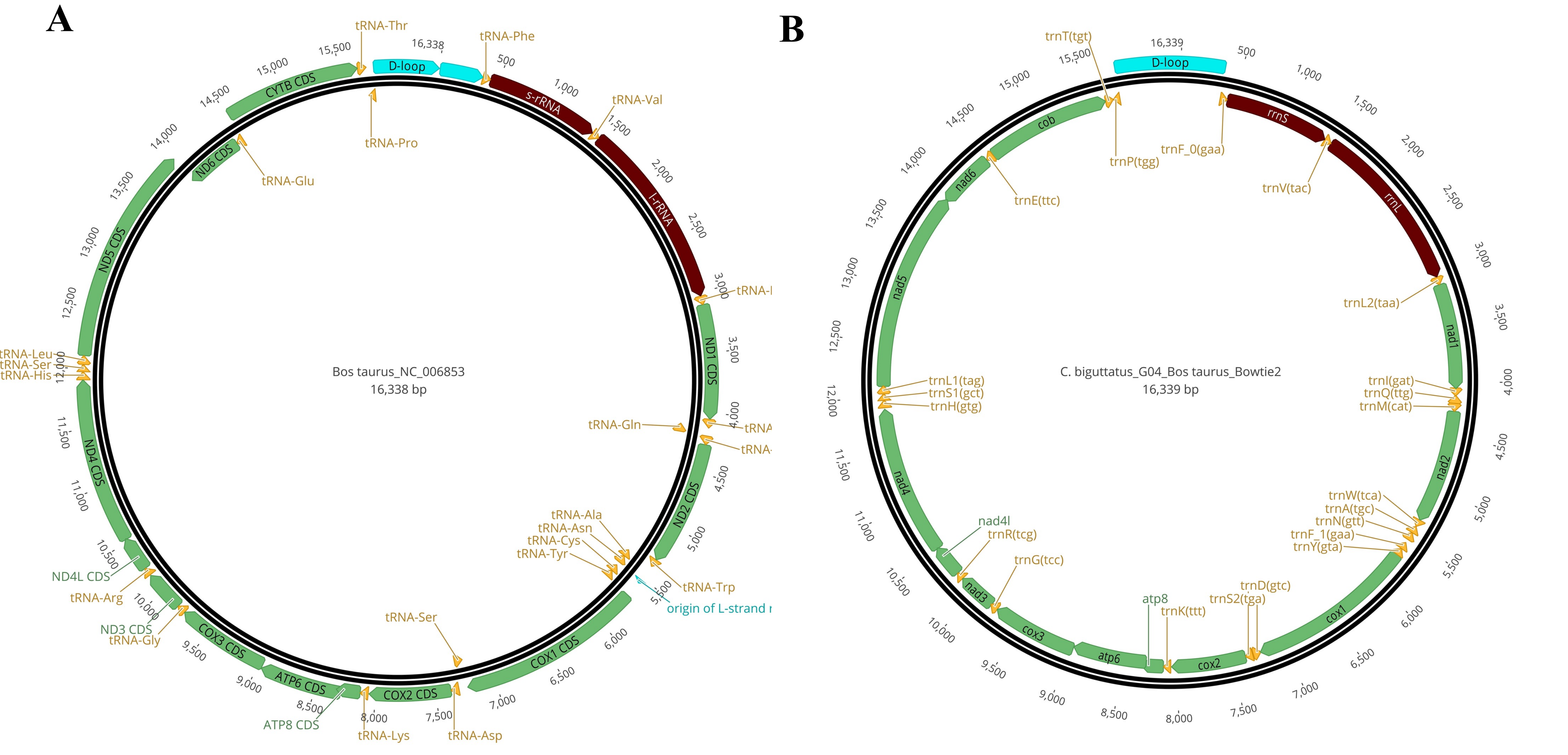
A
Bos taurus_NC_006853
16,338 bp
D-loop
s-rRNA
l-rRNA
ND1 CDS
ND2 CDS
COX1 CDS
COX2 CDS
ATP6 CDS
ATP8 CDS
COX3 CDS
ND3 CDS
ND4L CDS
ND4 CDS
ND5 CDS
ND6 CDS
CYTB CDS
tRNA-Thr
tRNA-Phe
tRNA-Pro
tRNA-Val
tRNA-Glu
tRNA-Gln
tRNA-Ala
tRNA-Asn
tRNA-Cys
tRNA-Tyr
tRNA-Trp
origin of L-strand
tRNA-Ser
tRNA-Lys
tRNA-Asp
tRNA-Gly
tRNA-Arg
tRNA-Leu
tRNA-Ser
tRNA-His
B
C. biguttatus_G04_Bos taurus_Bowtie2
16,339 bp
D-loop
rrnS
rrnL
nad1
nad2
cox1
cox2
atp8
atp6
cox3
nad3
nad4l
nad4
nad5
nad6
cob
trnT(tgt)
trnP(tgg)
trnF_0(gaa)
trnV(tac)
trnE(ttc)
trnL2(taa)
trnI(gat)
trnQ(ttg)
trnM(cat)
trnW(tca)
trnA(tgc)
trnN(gtt)
trnF_1(gaa)
trnY(gta)
trnD(gtc)
trnS2(tga)
trnK(ttt)
trnG(tcc)
trnR(tcg)
trnL1(tag)
trnS1(gct)
trnH(gtg)

Supplementary Figure S1. *Bos taurus* mitogenome recovered from *Culicoides biguttatus*. **A.** Reference mitogenome for *Bos taurus* (NC_006853) and **B.** *Bos taurus* mitogenome generated from *C. biguttatus*_G04 containing blood meal. PCGs, rRNA, and tRNA are indicated in green, brown, and orange. The control region (D-loop) is noted in blue.
