# Supplementary material for: Mitochondrial genome sequencing, mapping, and assembly benchmarking for Culicoides species (Diptera: Ceratopogonidae)
Source: BMC Genomics. 2022 Aug 13;23:584. doi: 10.1186/s12864-022-08743-x (PMC9375341; doi:10.1186/s12864-022-08743-x)
Supplement: Supplementary file 4 — Additional file 4: Supplementary Figure S2. Bayesian reconstruction of the relationship among Culicoides mitogenomes generated up to date, including current haplotypes. A. Blue square indicates C. biguttatus haplotypes mapped against C. arakawae. B. The red square indicates C. biguttatus haplotypes mapped against C. sonorensis_Scaffold710 using the same mapper (Bowtie2). Notice the switch in position of C. biguttatus depending on the reference mitogenome used. The numbers over the nodes indicate posterior probability support. Aedes aegypti (GenBank accession number NC_035159.1) represents the outgroup. [file 12864_2022_8743_MOESM4_ESM.pdf]

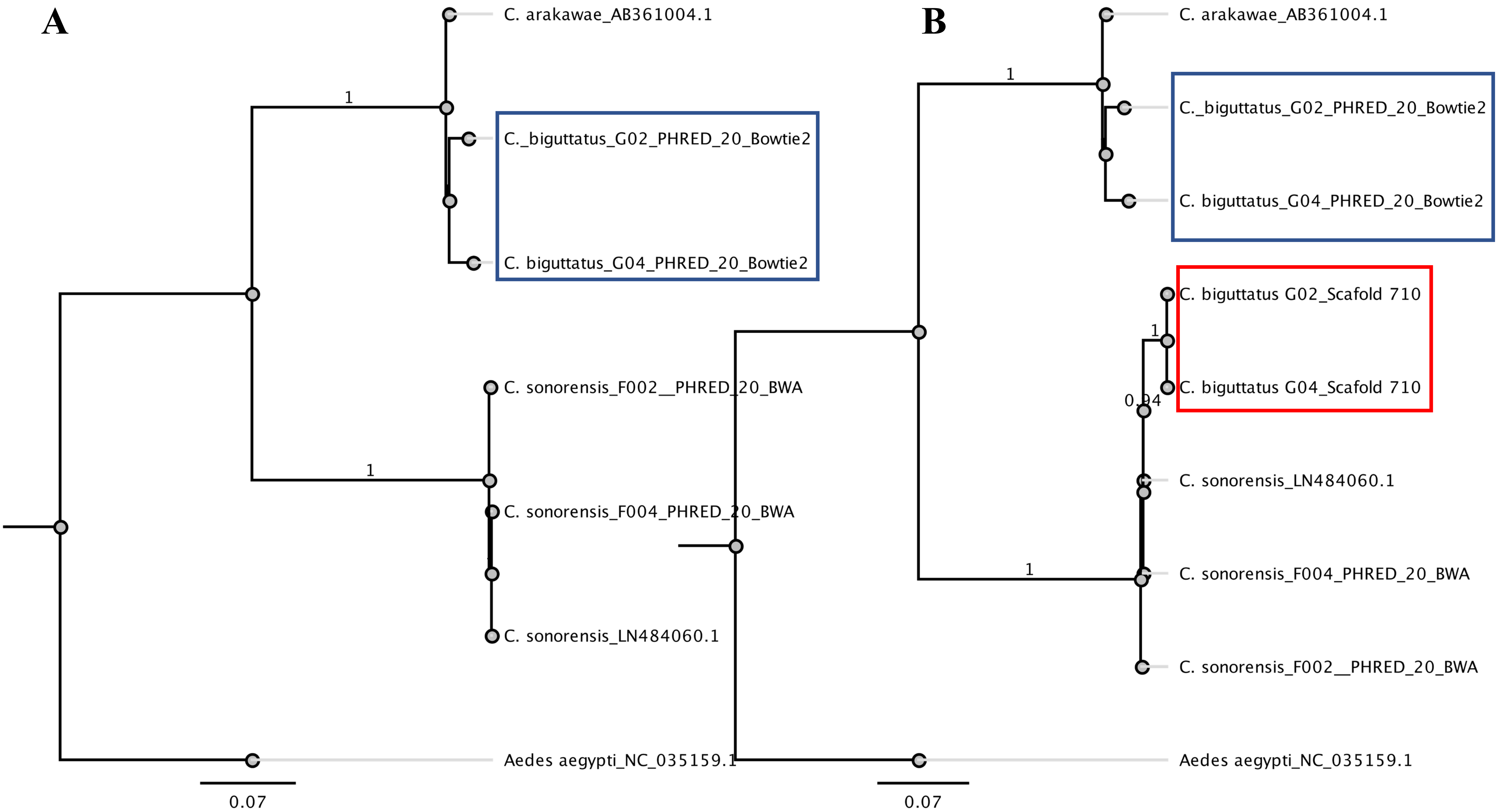

A
C. arakawae_AB361004.1
1
C._biguttatus_G02_PHRED_20_Bowtie2
C. biguttatus_G04_PHRED_20_Bowtie2
C. sonorensis_F002__PHRED_20_BWA
1
C. sonorensis_F004_PHRED_20_BWA
C. sonorensis_LN484060.1
Aedes aegypti_NC_035159.1
0.07
B
C. arakawae_AB361004.1
1
C._biguttatus_G02_PHRED_20_Bowtie2
C. biguttatus_G04_PHRED_20_Bowtie2
C. biguttatus G02_Scafold 710
1
C. biguttatus G04_Scafold 710
0.94
C. sonorensis_LN484060.1
1
C. sonorensis_F004_PHRED_20_BWA
C. sonorensis_F002__PHRED_20_BWA
Aedes aegypti_NC_035159.1
0.07

Supplementary Figure S2. Bayesian reconstruction of the relationship among *Culicoides* mitogenomes generated up to date, including current haplotypes. **A.** Blue square indicates *C. biguttatus* haplotypes mapped against *C. arakawae*. **B.** The red square indicates *C. biguttatus* haplotypes mapped against *C. sonorensis*_Scaffold710 using the same mapper (Bowtie2). Notice the switch in position of *C. biguttatus* depending on the reference mitogenome used. The numbers over the nodes indicate posterior probability support. *Aedes aegypti* (GenBank accession number NC_035159.1) represents the outgroup.
