# Supplementary material for: Mitochondrial genome sequencing, mapping, and assembly benchmarking for Culicoides species (Diptera: Ceratopogonidae)
Source: BMC Genomics. 2022 Aug 13;23:584. doi: 10.1186/s12864-022-08743-x (PMC9375341; doi:10.1186/s12864-022-08743-x)
Supplement: Supplementary file 5 — Additional file 5: Supplementary Figure S3. Heat map representing pairwise p genetic distances among Culicoides mitogenomes haplotypes available up to date. [file 12864_2022_8743_MOESM5_ESM.pdf]

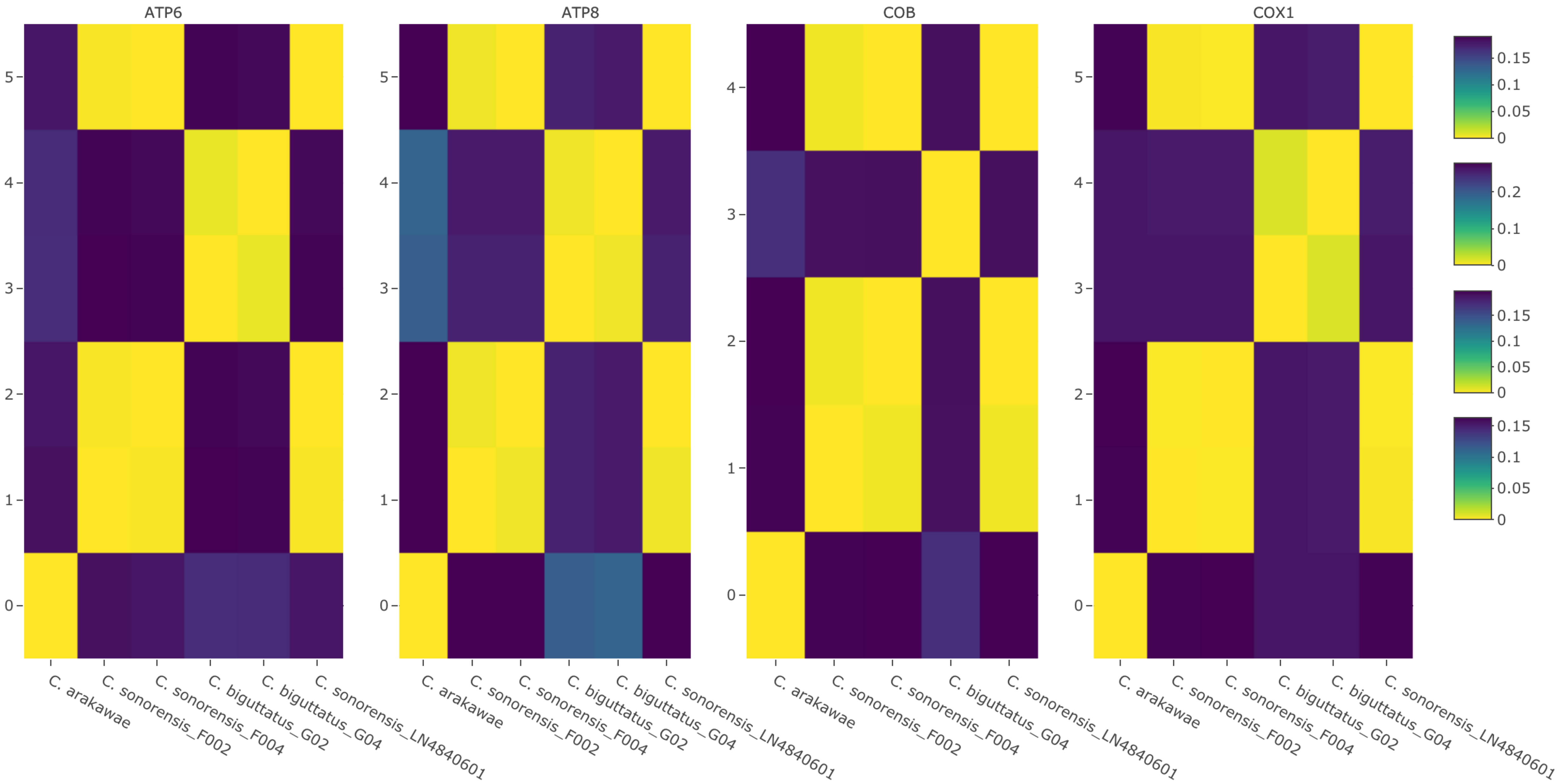

ATP6
ATP8
COB
COX1
0
1
2
3
4
5
C. arakawae
C. sonorensis_F002
C. sonorensis_F004
C. biguttatus_G02
C. biguttatus_G04
C. sonorensis_LN4840601
0.15
0.1
0.05
0
0.2
0.1
0

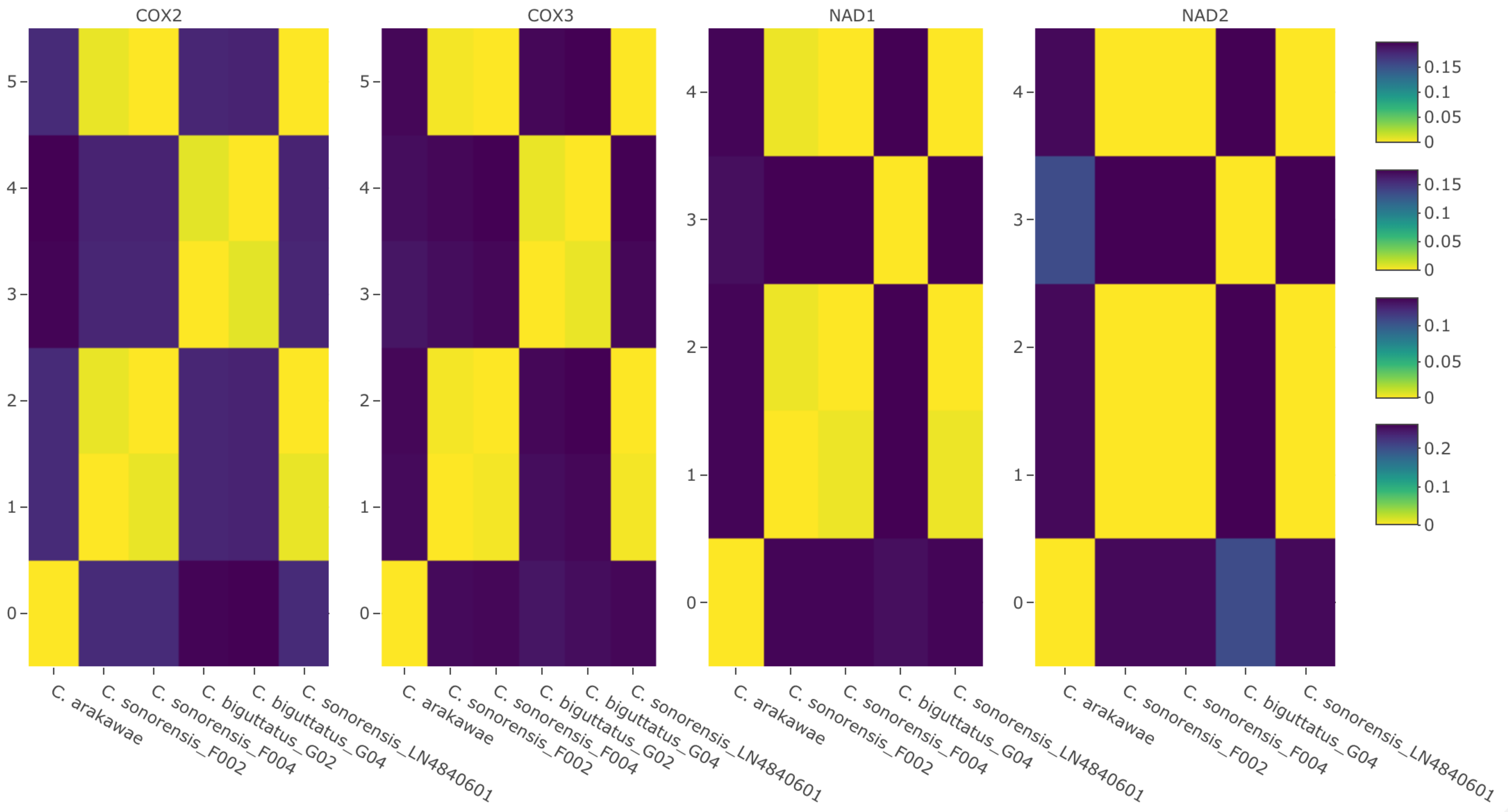

COX2
COX3
NAD1
NAD2
C. arakawae
C. sonorensis_F002
C. sonorensis_F004
C. biguttatus_G02
C. biguttatus_G04
C. sonorensis_LN4840601
0.15
0.1
0.05
0
0.2

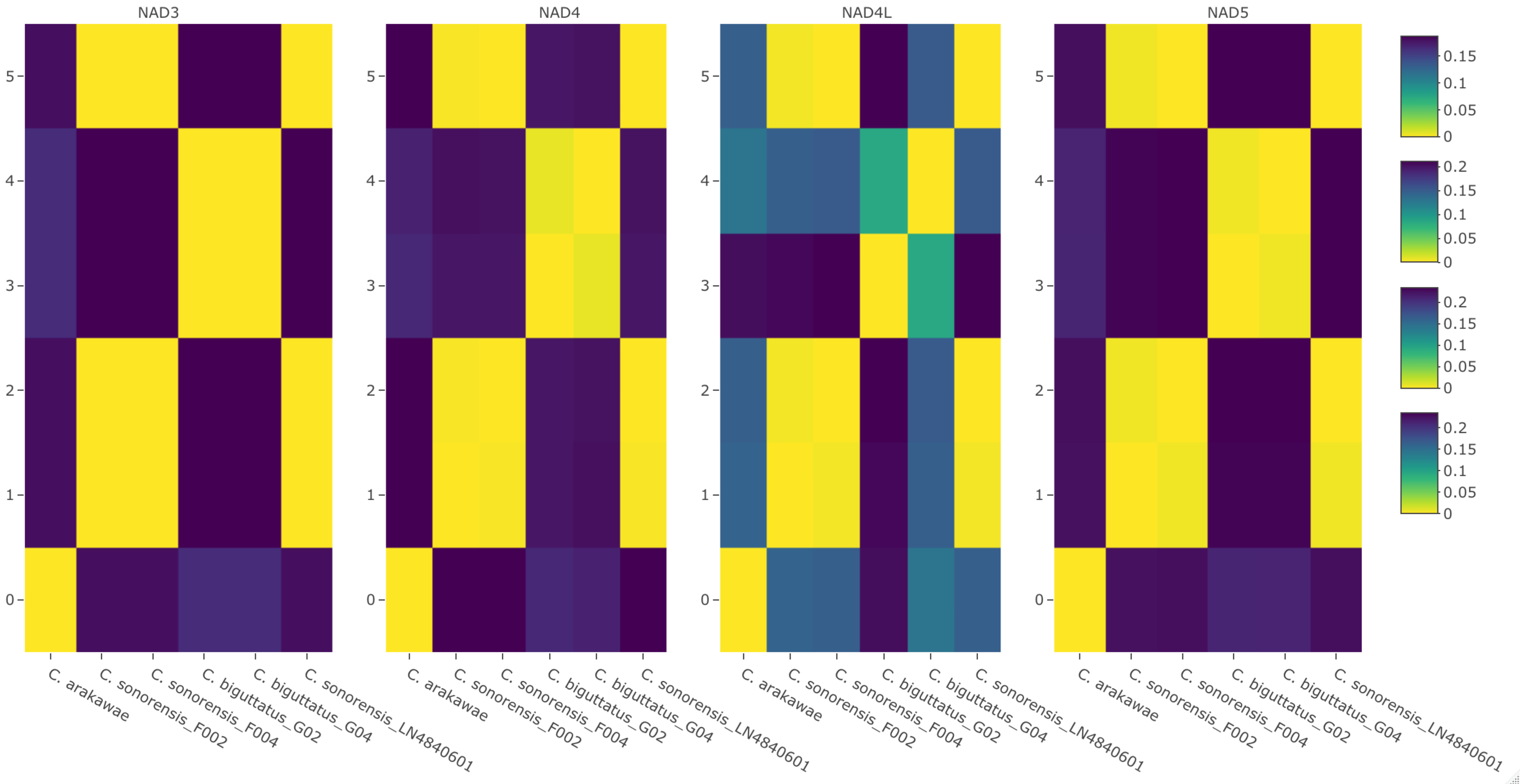
NAD3
NAD4
NAD4L
NAD5
0
1
2
3
4
5
C. arakawae
C. sonorensis_F002
C. sonorensis_F004
C. biguttatus_G02
C. biguttatus_G04
C. sonorensis_LN4840601
0
0.05
0.1
0.15
0.2

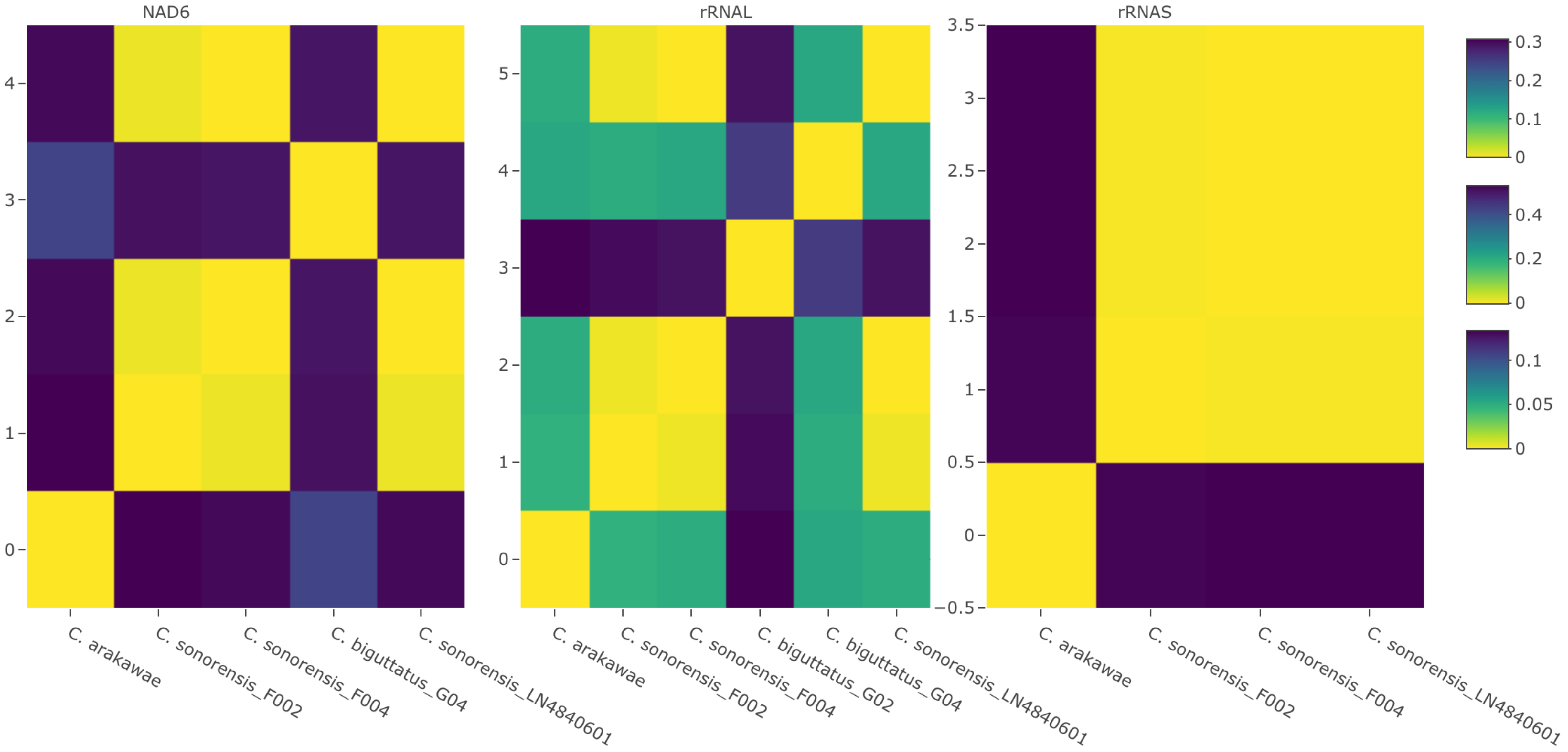

NAD6
rRNAL
rRNAS
4
3
2
1
0
5
3.5
3
2.5
2
1.5
1
0.5
0
−0.5
C. arakawae
C. sonorensis_F002
C. sonorensis_F004
C. biguttatus_G04
C. sonorensis_LN4840601
C. biguttatus_G02
0.3
0.2
0.1
0
0.4
0.2
0
0.1
0.05
0

Supplementary Figure S3. Heat map representing pairwise p genetic distances among *Culicoides* mitogenomes haplotypes available up to date.
